# Genome Analysis and Potential Ecological Functions of Members of the Genus *Ensifer* from Subsurface Environments and Description of *Ensifer oleiphilus* sp. nov.

**DOI:** 10.3390/microorganisms11092314

**Published:** 2023-09-14

**Authors:** Alexey P. Ershov, Tamara L. Babich, Denis S. Grouzdev, Diyana S. Sokolova, Ekaterina M. Semenova, Alexander N. Avtukh, Andrey B. Poltaraus, Elena A. Ianutsevich, Tamara N. Nazina

**Affiliations:** 1 Winogradsky Institute of Microbiology, Research Center of Biotechnology of the Russian Academy of Sciences, 119071 Moscow, Russia; e.alexey.mail@yandex.ru (A.P.E.); microb101@yandex.ru (T.L.B.); sokolovadiyana@gmail.com (D.S.S.); semenova_inmi@mail.ru (E.M.S.); e.a.ianutsevich@gmail.com (E.A.I.); 2 SciBear OU, 10115 Tallinn, Estonia; denisgrouzdev@gmail.com; 3 Skryabin Institute of Biochemistry and Physiology of Microorganisms, Russian Academy of Sciences, Pushchino Scientific Center for Biological Research of the Russian Academy of Sciences, 142290 Pushchino, Russia; avtukh@rambler.ru; 4 Engelhardt Institute of Molecular Biology, Russian Academy of Sciences, 119991 Moscow, Russia; abpolt@gmail.com

**Keywords:** genomics, *Ensifer*, oil formation water, radionuclide-contaminated groundwater, heavy-metal resistance, hydrocarbon degradation, nitrate reduction, *Ensifer oleiphilus*

## Abstract

The current work deals with genomic analysis, possible ecological functions, and biotechnological potential of two bacterial strains, HO-A22^T^ and SHC 2-14, isolated from unique subsurface environments, the Cheremukhovskoe oil field (Tatarstan, Russia) and nitrate- and radionuclide-contaminated groundwater (Tomsk region, Russia), respectively. New isolates were characterized using polyphasic taxonomy approaches and genomic analysis. The genomes of the strains HO-A22^T^ and SHC 2-14 contain the genes involved in nitrate reduction, hydrocarbon degradation, extracellular polysaccharide synthesis, and heavy metal detoxification, confirming the potential for their application in various environmental biotechnologies. Genomic data were confirmed by cultivation studies. Both strains were found to be neutrophilic, chemoorganotrophic, facultatively anaerobic bacteria, growing at 15–33 °C and 0–1.6% NaCl (*w*/*v*). The 16S rRNA gene sequences of the strains were similar to those of the type strains of the genus *Ensifer* (99.0–100.0%). Nevertheless, genomic characteristics of strain HO-A22^T^ were below the thresholds for species delineation: the calculated average nucleotide identity (ANI) values were 83.7–92.4% (<95%), and digital DNA–DNA hybridization (dDDH) values were within the range of 25.4–45.9% (<70%), which supported our conclusion that HO-A22^T^ (=VKM B-3646^T^ = KCTC 92427^T^) represented a novel species of the genus *Ensifer*, with the proposed name *Ensifer oleiphilus* sp. nov. Strain SHC 2-14 was assigned to the species ‘*Ensifer canadensis*’, which has not been validly published. This study expanded the knowledge about the phenotypic diversity among members of the genus *Ensifer* and its potential for the biotechnologies of oil recovery and radionuclide pollution treatment.

## 1. Introduction

The taxonomic position of the bacterial species of the genera *Ensifer* and *Sinorhizobium* has been repeatedly revised over the past two decades [1,2]. These bacteria form a single phylogenetic group within the family *Rhizobiaceae*, referred to as the *Ensifer*/*Sinorhizobium* clade. A number of suggestions have been made to transfer the previously described *Ensifer* species to the genus *Sinorhizobium* and vice versa [3,4]. Recent data revealed that despite the high phylogenetic similarity of all detected strains (cpAAI > 86%), some species from the clade differ significantly from the rest in their biology and ecology [5]. This fact has made it possible to separate these genera among themselves, to assign 17 validly described bacterial species to the genus *Sinorhizobium* and the species *E. adhaerens*, *E. morelensis*, *E. sesbaniae*, and ‘*E. canadensis*’ to the genus *Ensifer* [5,6]. Members of the genus *Ensifer*, originally described by Casida [7], are budding bacteria, able to grow in LB medium and to hydrolyze starch, with an optimal temperature at 27–28 °C; they are generally nonsymbiotic (except for symbiosis of *E. sesbaniae* with legumes and that of ‘*E. canadensis*’ with roots of *Melilotus albus*), slightly acidophilic, and resistant to ampicillin and erythromycin [5,6]. Some members of the genus *Ensifer* are applicable in biotechnologies of xenobiotics degradation [8,9].

Two bacterial strains, HO-A22^T^ and SHC 2-14, were isolated from an oil field water and radionuclide-contaminated groundwater (Russia), respectively. Microorganisms isolated from petroleum reservoirs are of great potential for application in microbial enhancement of oil recovery (MEOR) technologies. Some of these technologies are based on the production of exopolysaccharides by the reservoir microbiota [10], which leads to the redirection of injected water flows and, as a result, an increase in the coverage of the oil reservoir by flooding. Several bacterial strains from the *Ensifer*/*Sinorhizobium* clade are known to release significant amounts of exopolysaccharides into the environment [11,12,13,14], and,, therefore may be useful for MEOR technologies in oil fields. Moreover, injection of nitrate into petroleum reservoirs can activate the growth of denitrifying bacteria, which leads to suppression of the sulfidogenic microbiota and, therefore, decreases the risks of oil field equipment biocorrosion [15,16,17]. Isolates of the genera *Ensifer* and *Sinorhizobium* are known to reduce nitrate ions during their growth [18,19,20], making it potentially possible to stimulate bacteria of this group in the formation water in order to control the petroleum reservoir souring.

Bacteria of the genera *Ensifer* and *Sinorhizobium*, capable of denitrification, can affect the mobility of variable-valency metals and radionuclides in groundwater contaminated with components of liquid radioactive waste in the areas of the nuclear fuel cycle enterprises. When liquid radioactive waste is buried in surface water bodies and underground layers, toxic components gradually seep into the groundwater [21,22]. Liquid radioactive wastes are enriched in nitrates and organic salts. In the process of denitrification, due to the reduction of nitrates to molecular nitrogen, the ambient redox potential decreases [23], which leads to the transition of radionuclides and heavy metals into a poorly soluble reduced form. This process can be used to create a geochemical barrier for radionuclides in groundwater contaminated with toxic components of liquid radioactive waste.

The microorganisms can also have an effect on radionuclides and heavy metals due to their immobilization in the exopolysaccharide matrix of bacterial biofilms [23,24], in the processes of biosorption and bioaccumulation on the surface or inside the cells [25,26], and in the processes of direct reduction of variable-valency elements to poorly soluble compounds during cellular respiration [22,24,27,28,29]. Thus, the study of microorganisms isolated from radionuclide-contaminated groundwater, as well as from oil reservoirs, has both basic and applied importance.

The aim of our study was to estimate the biotechnological potential of strains HO-A22^T^ and SHC 2-14 belonging to the genus *Ensifer* based on its genomic and phenotypic characterization and taxonomic description of the strain HO-A22^T^ as *Ensifer oleiphilus* sp. nov. The study of the strain SHC 2-14 allowed us to assign it to the not validly published species ‘*Ensifer canadensis*’ and to expand knowledge about the phenotypic diversity of this species. The ecological values of the isolated strains and their potential for the biotechnologies associated with oil recovery and radionuclide pollution treatment were also discussed.

## 2. Materials and Methods

### 2.1. Sources of Isolation of Bacterial Strains

Strain HO-A22^T^ was isolated from a sample of injection water represented by a mixture of fresh river water and production water reinjected into the Cheremukhovskoe oil field (Tatarstan, Russia). The water sample was collected in 2016 during a field experiment of a microbial enhanced oil recovery (MEOR) technology. In the terrigenous Cheremukhovskoe heavy oil reservoir, formation water belonged to the chlorine-calcium type. pH of the water sample was 7.86, and its density (at 25 °C) was 997.4 kg·m^–3^. The injection water contained the following dissolved ions (mg·L^–1^): hydrocarbonate (244), chloride (114), potassium and sodium (114), sulfate (74), calcium (60), and magnesium (15). The total salinity of the water sample was 620 mg·L^–1^. The depth of the reservoir was 850–1300 m below sea level, and its average temperature was 22–25 °C. Other physicochemical characteristics of the water sample were described by Nazina et al. [30]. 

Strain SHC 2-14 was isolated from a groundwater sample collected in 2015 through an observation well from an upper water-bearing horizon at the depth of 10 m near a preserved surface repository for radioactive waste (Tomsk region, Russia) [21]. The temperature of the horizon was 6–8 °C; the groundwater sample had total salinity of 7 g·L^–1^ and pH 5.9. Redox potential of the groundwater varied from –20 to +150 mV (from +50 to +70 mV on average), so it might be characterized as microaerobic. The sample contained dissolved ions at the following concentrations (mg·L^–1^): nitrate (4239), calcium (772), sodium (570), hydrocarbonate (170), magnesium (146), carbonate (135), sulfate (133), etc. The presence of significant amounts of strontium, cobalt, cesium, and plutonium in the sample was observed.

Strains HO-A22^T^ and SHC 2-14 were cultivated on the TEG medium containing bacto-trypton (5.0 g), yeast extract (2.5 g), glucose (1.0 g), NaCl (5.0 g), and distilled water (1 L, pH 7.0–7.2). All reagents were purchased from Dia-M (Moscow, Russia) and were chemically pure. Isolation of the strains was performed by serial dilution technique as described before [29]. To obtain pure cultures, the medium was supplemented with bacteriological agar (20 g·L^–1^), and then single colonies were reinoculated in the liquid broth. The strains have been deposited at the All-Russian Collection of Microorganisms (VKM; Pushchino, Moscow Region, Russia) and at the Korean Collection for Type Cultures (KCTC; Jeongeup-si, Jeollabuk-do, South Korea) under the numbers VKM B-3646^T^ and KCTC 92427^T^ (strain HO-A22^T^) and VKM B-3385 and KCTC 92426 (strain SHC 2-14). For comparative studies, type strains *E. adhaerens* A^T^ (=NBRC 100388^T^ = LMG 20216^T^ = ATCC 33212^T^) and *E. morelensis* Lc04^T^ (=NBRC 100387^T^ = LMG 21331^T^ = CFN E1007^T^) were obtained from the Biological Resource Center, NITE (NBRC), Tokyo, Japan.

### 2.2. DNA Extraction, 16S rRNA Gene Sequencing and Phylogenetic Analysis

The DNA of the strains was extracted from bacterial biomass using Diatom™ DNA Prep 100 (Isogen Laboratory, Moscow, Russia) according to the manufacturer’s recommendations. The purified DNA samples were used as the PCR templates. Pure cultures of bacteria were identified by 16S rRNA gene analysis using the Bacteria-universal primer set 27F–1492R [31]. DNA sequencing was performed according to the Sanger method on an ABI 3730 DNA Analyzer automatic sequencer using the ABI PRISM^®^ BigDye™ Terminator v. 3.1 reagent kit (Applied Biosystems, Waltham, MA, USA).

Initial taxonomic assignment of strains HO-A22^T^ and SHC 2-14 was carried out using EzBioCloud [32]. The phylogenetic position of the isolated strains was determined using 16S rRNA gene sequences of type strains of the family *Rhizobiaceae*. The 16S rRNA gene sequences were aligned by MUSCLE [33], and the maximum-likelihood tree was performed with a GTR+F+I+G4 model recommended by ModelFinder [34] in IQ-TREE [35]. Branch supports were obtained with 10,000 ultrafast bootstraps [36]. Maximum parsimony and neighbor-joining trees were reconstructed with MPBoot [37] and MEGA11 [38], respectively.

### 2.3. Genome Sequencing and Analyses

Genomic DNA for the sequencing was extracted from bacterial biomass using the QIAamp DNA Mini Kit (QIAGEN, Germantown, MD, USA). The DNA libraries were constructed with the NEBNext DNA library prep reagent set for Illumina, according to the protocol for the kit. Sequencing of genomic DNA was carried out using the HiSeq 2500 platform (Illumina, Inc., San Diego, CA, USA) with 150-bp paired-end reads. The raw reads were quality checked with FastQC v. 0.11.9 (http://www.bioinformatics.babraham.ac.uk/projects/fastqc/ (accessed on 8 January 2019), Version 0.12.0 released 1 March 2023), and low-quality reads were trimmed with Trimmomatic v. 0.39 [39], using the default settings for paired-end reads. The quality-filtered reads were then de novo assembled with SPAdes v. 3.13.0 [40] using the default settings. The resulting assembly was quality assessed with QUAST v. 5.0 [41]. The genome coverages were estimated by QualiMap 2 v. 2.2.2 [42] and Bowtie 2 v. 2.3.5.1 [43]. Genome annotation was performed using the NCBI Prokaryotic Genome Annotation Pipeline (PGAP; v. 4.7) [44].

Complete genomic sequences were analyzed to confirm the assignment of new strains at the species and genus levels as recommended by Chun et al. [45]. Phylogenetic analysis of the isolated strains and members of the family *Rhizobiaceae* was performed based on a concatenated alignment of 120 single-copy genes obtained using GTDB-Tk software version 1.0.2 [46]. The maximum likelihood phylogenetic tree was calculated using IQ-TREE [35], based on the ModelFinder recommendations [34], and the branching support was estimated using UFBoot2 [36]. MPBoot [37] and MEGA11 [38] were used to reconstruct the maximum parsimony and neighbor-joining trees, respectively. DNA–DNA hybridization (dDDH) and average nucleotide identity (ANI) values were determined using Genome-to-Genome Distance Calculator (GGDC) v. 3.0 [47,48] and FastANI v. 1.3 [49], respectively. Average amino acid identity (AAI) was calculated using CompareM v. 0.0.23 with the aai_wf function. POCP values were calculated with the script runPOCP.sh [50] based on the previously described approach [51]. The pangenomic analysis was performed based on a bioinformatic pipeline proposed [52] using Anvi’o version 7.0 [53]. Using the MCL algorithm (Euclidean distance, Ward linkage), genomes were arranged according to the distribution of gene clusters.

### 2.4. Phenotypic Characterization

TEG medium was used to investigate the morphological, physiological, and biochemical characteristics of the strains, including fatty acids profiles, phospholipids of the cell walls, and quinones. All physiological tests were carried out in triplicate and chemotaxonomic tests in duplicate. Growth at different temperatures (5, 9, 15, 22, 28, 30, 33, 37, and 42 °C), salt tolerance (0, 0.1, 0.3, 0.5, 0.8, 1.2, 1.6, 2.0, 2.5, 3.0, 3.5, and 4.0% (*w*/*v*) NaCl) and pH range for growth (5.0–10.5 with increments of ~0.5 pH units) were determined on TEG medium for 3–5 days at aerobic conditions optimal for growth of the strains.

Motility of the studied cells was estimated under an Olympus CX41 system microscope (Tokyo, Japan) with a phase contrast device and a 100× objective with oil immersion. For the scanning electron microscopy, biomass of the isolated strains was grown in a TEG medium with Teflon cubes and then was prepared for the observation using phosphate buffer (pH 7.0), ethyl alcohol, and acetone as described previously [54]. The samples were examined under a scanning electron microscope (Quattro S, “Thermo Fisher Scientific Brno s.r.o.”, Brno-Černovice, Czech Republic) under high vacuum at an accelerating voltage 15 kV.

Catalase activity was determined by the conventional method with hydrogen peroxide. Enzyme activities and substrates consumption by the studied strains were measured using API^®^ ZYM, API^®^ 20E, and API^®^ 50CH tests (bioMérieux SA, Marcy-l’Étoile, France) at 22 °C for 3–5 days. Antibiotics sensitivity was investigated on Petri dishes with susceptibility test discs (HiMedia, Mumbai, Maharashtra, India) with carbenicillin (100 μg), erythromycin (15 μg), gentamicin (10 μg), and kanamycin (30 μg), based on the presence of growth inhibition zones >10 mm around the discs after 5 days of cultivation.

To study utilization of oil alkanes, Adkins mineral medium [55] with pH 7.0–7.2 supplemented with a premade trace elements solution (10 mL·L^–1^) [56] was used. Sterilized oil from production well 5462 of the Cheremukhovskoe oil field was added at a concentration of 0.5% (*v*/*v*). To investigate the denitrifying activity of the strains, Adkins medium with argon as the gas phase was deoxygenated by boiling and supplemented with glucose (5.0 g·L^–1^), L-cysteine (0.5 g·L^–1^), and sodium nitrate (0.85 g·L^–1^).

### 2.5. Chemotaxonomic Characterization

The fatty acid composition was determined as the percentage of the total ion current peak area using a 7890B+5977B gas chromatograph-mass spectrometer (Agilent Technologies, Santa Clara, CA, USA). The biomass of the strains was dried with methanol and subjected to acidic methanolysis (1.2 M HCl/MeOH, 80 °C, 10 min) as described earlier [57]. The fatty acid composition was analyzed using a Maestro gas chromatograph-mass spectrometer (Interlab, Moscow region, Russia). The analysis of respiratory quinones of strains was performed at the All-Russian Collection of Microorganisms. Isoprenoid quinones were extracted from wet cells, purified according to Collins and Jones [58], and analyzed with an Thermo Finnigan LCQ Advantage MAX mass spectrometer (Thermo Fisher Scientific Inc., Waltham, MA, USA). Two-dimensional thin-layer chromatography on silica gel layers was performed for polar lipids identification [59]. Spraying with ninhydrin, α-naphthol, Dittmer–Lester molybdenum blue reagent, and Dragendorff reagent was used for visualization of aminolipids, glycolipids, phospholipids, and choline, respectively, as described in detail previously [60].

### 2.6. Analytical Methods

The growth of biomass was monitored by the changes in its optical density at 660 nm using an Ultrospec 2100 pro spectrophotometer (Amersham Biosciences, Amersham, Buckinghamshire, UK). The pH of the cultivation media was measured with a Seven Compact S220 pH meter (Metter Toledo, Greifensee, Switzerland). Formation of N_2_ or/and N_2_O by nitrate-reducing bacteria was estimated using a Crystal 5000 gas chromatograph (Chromatec, Yoshkar-Ola, Mari El, Russia) with a HayeSep-N 80/100 column.

Rheological characteristics of the cultivation media, i.e., surface tension (between the medium and air) and interfacial tension (medium/*n*-hexadecane) were measured with a Surface Tensiomat 21 semi-automatic tensiometer (Cole-Parmer, Vernon Hills, IL, USA) by the ring separation method at 25 °C. To estimate the utilization of oil alkanes, nonpolar compounds of the cultivation media were extracted with *n*-hexane and fractionated on silica gel columns. The profiles of light aliphatic hydrocarbons were determined using a Crystal 5000.1 gas chromatograph (Chromatec, Yoshkar-Ola, Mari El, Russia) with a ZB-FFAP 15 m capillary column and a flame ionization detector. Evaluation of oil biodegradation by the studied strains and its internal normalization based on the *n*-alkanes to *iso*-alkanes ratio were performed as described earlier [61].

### 2.7. Bacterial Reduction of Radionuclides and Metals

The biomass of the strain SHC 2-14 was grown aerobically on the TEG medium and collected by centrifugation [22]. About 0.125 g·L^–1^ (dry weight) of the live biomass was incubated for 10 days anaerobically in physiological saline using (metal ion concentrations, mg·L^–1^) uranyl nitrate (50), sodium pertechnetate (10), potassium bichromate (30), sodium selenate (50), ammonium vanadate (50), or/and sodium molybdate (30) as electron acceptors, and sodium acetate (20 mM) or sodium lactate (30 mM) as electron and carbon donors. The oxidation state of U, Cr, Se, V, and Mo was determined using a Bruker Elexsys E580 pulse EPR spectrometer (Billerica, MA, USA) in 0.6 mL of the liquid phase. Tc(IV) was determined by X-ray photoelectron spectroscopy (XPS) analysis of the solid phase. The samples were centrifuged for 5 min; then the supernatant was decanted, dried at 105 °C, and milled. Milled samples were embedded into indium on an aluminum support plate in form of thin films with mirror-like surfaces. Each sample was measured under a vacuum of 5·10^–7^ Pa at 24 °C using the low-energy gun for the sample charge compensation. The XPS spectra were obtained using a Kratos Axis Ultra DLD (Kratos Analytical Ltd., Manchester, UK) spectrometer with AlKα monochromatized (1486.6 eV) X-ray source.

### 2.8. Nucleotide Sequence Accession Numbers

The GenBank/EMBL/DDBJ accession numbers for the 16S rRNA gene sequences of strains HO-A22^T^ and SHC 2-14 are MT495799 and MG051317, respectively. The GenBank/EMBL/DDBJ accession numbers of the genomic assemblies of strains HO-A22^T^ and SHC 2-14 are GCF_013371465.1 (JABWDU000000000.1) and GCF_013141885.1 (SROZ00000000.1), respectively.

## 3. Results and Discussion

### 3.1. Morphological Characterization of the Isolated Strains

**Figure 1:**
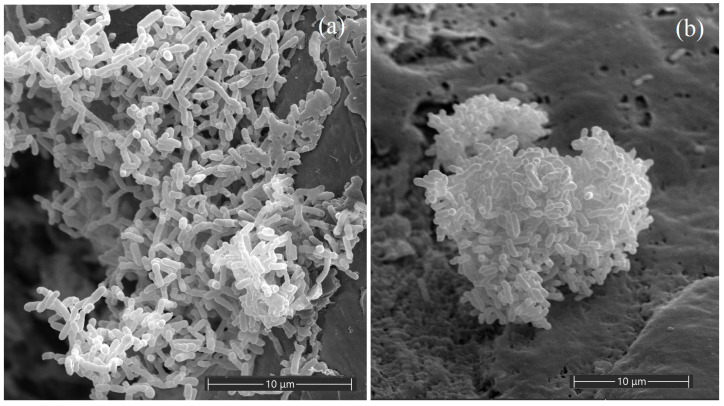
Cell morphology of the strains HO-A22^T^ (**a**) and SHC 2-14 (**b**) grown in TEG medium at 22 °C for 72 h. The samples were examined under a scanning electron microscope (Quattro S, “Thermo Fisher Scientific”, Brno-Černovice, Czech Republic) with accelerating voltage 15 kV. Bars, 10 µm.

To study the characteristics of the isolated strains, they were analyzed with morphological, genomic, physiological, and chemotaxonomic methods. Cells of the strains HO-A22^T^ and SHC 2-14 were found to be asporogenous, motile, Gram-stain negative rods, 1.0–1.5 × 0.3–0.5 μm and 1.3–2.2 × 0.6–0.9 μm, respectively (Figure 1). After 5 days of cultivation at 22 °C, the strains formed creamy, smooth, shiny, and round colonies on the medium, up to 8.0 and 6.0 mm in diameter, respectively. The purity of the isolated cultures was confirmed by light microscopy and by 16S rRNA gene analysis.

### 3.2. Analysis of the 16S rRNA Genes

**Figure 2:**
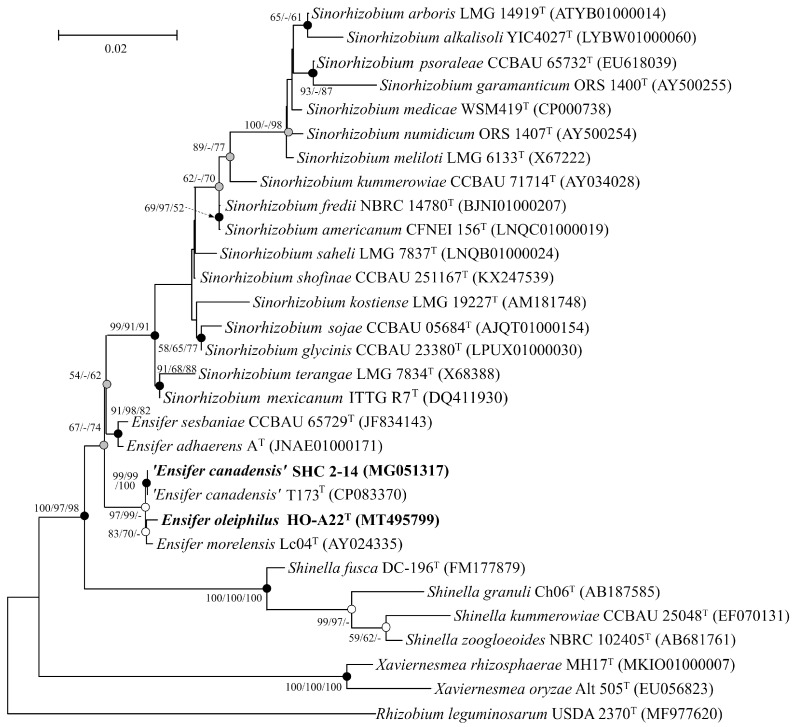
Maximum-likelihood phylogenetic tree based on 16S rRNA gene sequences (1365 nucleotide sites) reconstructed with evolutionary model GTR+F+I+G4, showing the position of strains HO-A22T and ‘*E. canadensis*’ SHC 2-14 with closely related members of the family *Rhizobiaceae*. Grey circles indicate that the corresponding nodes were recovered in the reconstructed tree based on the maximum-parsimony algorithm; white circles indicate that the corresponding nodes were recovered using the neighbor-joining algorithm; black circles indicate that the corresponding nodes were also recovered based on the neighbor-joining and maximum-parsimony algorithms. Bootstrap values (>50%) are listed as percentages at the branching points. Bar, 0.02 substitutions per nucleotide position. The tree was rooted using *Rhizobium leguminosarum* USDA 2370^T^ as the outgroup. GenBank accession numbers for 16S rRNA genes are indicated in brackets.

The 16S rRNA gene sequence similarity between new strains was 99.8%, indicating that they could probably belong to the same species [45]. The 16S rRNA genes of strains HO-A22^T^ and SHC 2-14 showed the highest similarity to those of the members of the genera *Ensifer* (99.0–100.0%) and *Sinorhizobium* (97.4–98.8%) of the family *Rhizobiaceae*. On the phylogenetic tree (Figure 2), the 16S rRNA gene sequences of the strains HO-A22^T^ and SHC 2-14 formed a branch with that of *Ensifer morelensis* Lc04^T^ [4]. At the time of writing this article, a new species ‘*Ensifer canadensis*’ has been described, also belonging to the *E. morelensis* Lc04^T^ branch [6]. The tree had low bootstrap values, which indicated the need for phylogenetic analysis. Nevertheless, the results of phylogenetic analysis of the 16S rRNA genes demonstrated that the new isolates belonged to the *Ensifer* genus. To clarify the taxonomic position of strains HO-A22^T^ and SHC 2-14, their morphological, physiological, and chemotaxonomic properties were studied, and their genomes were analyzed.

### 3.3. Phylogenetic Analysis

**Figure 3:**
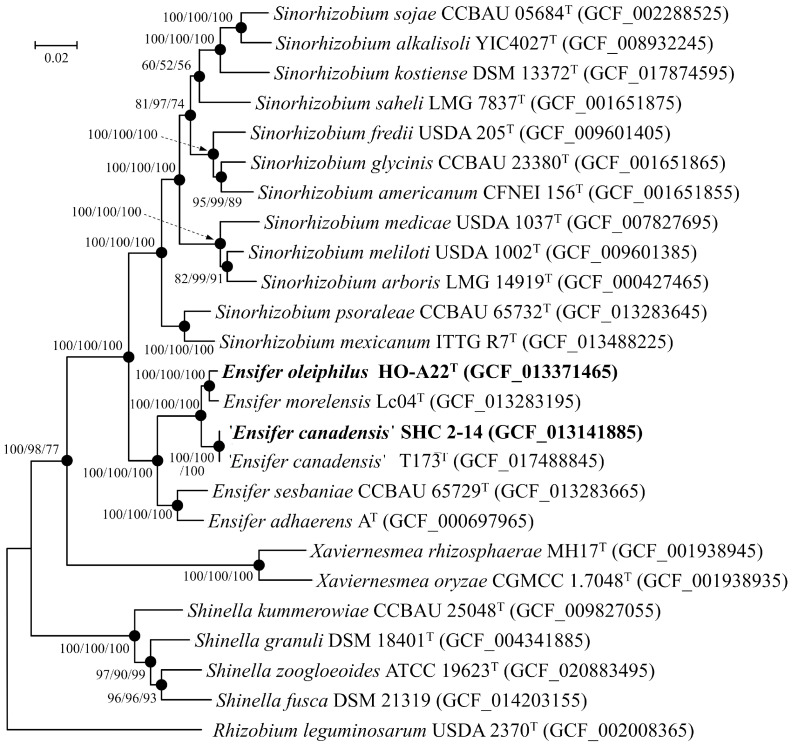
The maximum-likelihood phylogenetic tree derived from concatenated 120 single copy proteins shows the position of strains HO-A22^T^ and ‘*E. canadensis*’ SHC 2-14 in relation to closely related members of the family *Rhizobiaceae*. Phylogenetic analysis was performed with the LG+F+I+G4 model using 37,730 amino acid positions. Black circles indicate that the corresponding nodes were also recovered based on the neighbor-joining and maximum-parsimony algorithms. Bar, 0.02 amino acid substitutions per site. Bootstrap values (>50%) are listed as percentages at the branching points. The tree was rooted using *Rhizobium leguminosarum* USDA 2370^T^ as the outgroup. Accession numbers for the genomic assemblies are indicated in brackets.

The strain HO-A22^T^ genome consists of 6,762,417 bp and comprises 32 scaffolds, an N_50_ value of 1,120,613 bp, a G+C content of 61.7%, and coverage of 225×. The strain SHC 2-14 genome consists of 8,169,716 bp and comprises 33 scaffolds, an N_50_ value of 332,742 bp, a G+C content of 61.0%, and coverage of 165×. Annotation led to the identification of 6343 genes, including 6114 protein-coding sequences, 174 pseudogenes, and 55 RNA genes for strain HO-A22^T^ and 7808 genes, including 7411 protein-coding sequences, 340 pseudogenes, and 57 RNA genes for strain SHC 2-14. On the phylogenetic tree, strains HO-A22^T^ and SHC 2-14 formed a branch with the type strains of *E. morelensis*, *E. adhaerens*, *E. sesbaniae*, and ‘*E. canadensis*’ (Figure 3). This branch was located at a considerable distance from that of the *Sinorhizobium* type species. The calculated AAI and POCP values between new strains and the analyzed *Ensifer* strains were 83.7–99.1% and 74.1–88.7%, respectively (Table 1). These values for the strain HO-A22^T^ exceed the thresholds for identifying bacteria as a separate genus (AAI for the family *Rhizobiaceae* < 76.5% [5] and POCP < 50–60% [51,62,63,64]. These results confirm the data of phylogenetic analysis of the 16S rRNA gene sequences, indicating that the strains HO-A22^T^ and SHC 2-14 belong to the *Ensifer* genus.

**Figure 4:**
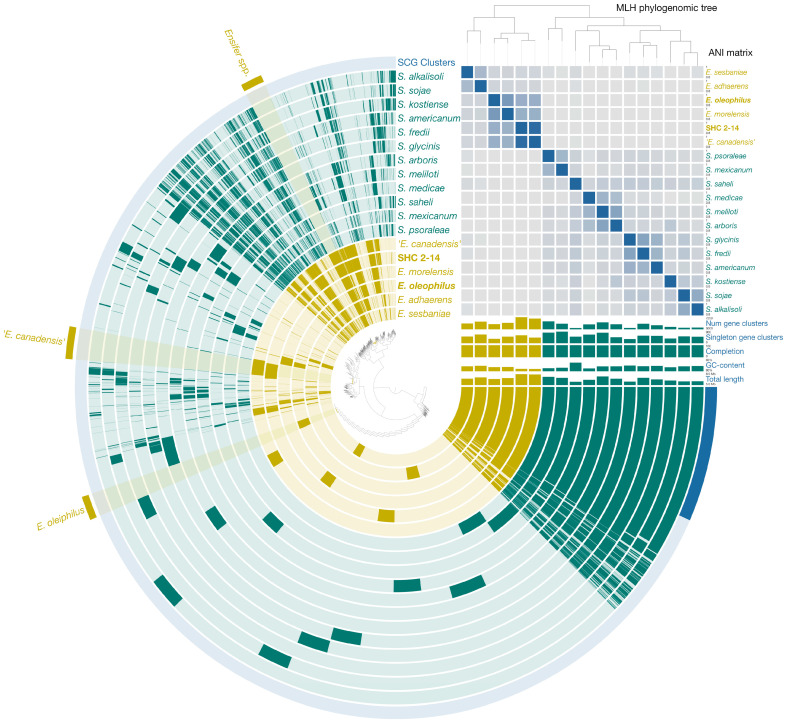
Pangenome analysis of *Ensifer* and *Sinorhizobium* type strains calculated with Anvi’o v. 7.0. Central dendrogram represents relationships between 22,072 gene clusters (117,350 genes) in all analyzed genomes. Dark circular regions represent genes found in those areas for each genome. The phylogenetic tree is reconstructed using the single copy genes. ANI heatmap in blue squares varies between 70 and 100%.

Pangenomic analysis was performed on 18 genomes from the *Ensifer*/*Sinorhizobium* clade. The resulting pangenome consisted of 117,350 genes grouped into 22,072 gene clusters (GCs). Among these clusters, 2502 were identified as core clusters present in all analyzed genomes, indicating their significance for the clade (Figure 4). 

Core clusters included the genes involved in amino acid transport and metabolism (291 GCs) and translation, ribosomal structure, and biogenesis (211 GCs). The analysis revealed 207 GCs responsible for complete pathways of carbohydrate metabolism, encompassing glycolysis (Embden–Meyerhof pathway), Entner–Doudoroff pathway, pyruvate oxidation, citrate cycle (TCA cycle), pentose phosphate cycle, PRPP biosynthesis, UDP-N-acetyl-D-glucosamine biosynthesis, and the exo cluster for succinoglycan-type exopolysaccharide biosynthesis and production. Additionally, 101 GCs involved in energy metabolism were identified, encompassing genes of NADH:quinone oxidoreductase, succinate dehydrogenase, cytochrome bc1 complex, cytochrome c oxidase, and F-type ATPase. The genomes also contained the genes for assimilatory nitrate and sulfate reduction.

Comparing *Ensifer* and *Sinorhizobium* genomes, 313 GCs were found to be shared by all *Ensifer* genomes but not in *Sinorhizobium* genomes. These included *curA* (NADPH-dependent curcumin reductase), *cysP* (sulfate/thiosulfate transport system substrate-binding protein), *gli* (D-galactarolactone isomerase), *uxaB* (tagaturonate reductase), *yphE* and *yphF* (sugar transport system genes), *adeS* (sensor histidine kinase), *lplA*, *lplB*, and *lplC* (putative aldouronate transport system genes), as well as *ceuA*, *ceuC*, and *ceuD* (iron-siderophore transport system genes).

The genome of strain HO-A22^T^ possessed 339 GCs unique to the *Ensifer*/*Sinorhizobium* clade, 75 of which were annotated and involved in transcription, signal transduction mechanisms, amino acid metabolism, and inorganic ion transport and metabolism. The pangenome of ‘*E. canadensis*’ SHC 2-14 and ‘*E. canadensis*’ T173^T^ consisted of 15,524 genes grouped into 8004 GCs. These genomes harbored 451 unique for the ‘*E. canadensis*’ clusters, including the genes associated with heavy-metal resistance, such as those of arsenical pump membrane protein ArsB and P-type Cu^2+^ transporter CopB (EC: 7.2.2.9).

These findings demonstrate the genetic diversity within the *Ensifer*/*Sinorhizobium* clade and provide insights into the adaptations of the studied strains to their respective habitats.

### 3.4. Genomic Analysis and Environmental Implications

The genomes of strains HO-A22^T^ and ‘*E. canadensis*’ SHC 2-14 both possess numerous carbon metabolism genes, including those for glycolysis (Embden–Meyerhof pathway), gluconeogenesis, TCA cycle, pentose phosphate and Entner–Doudoroff pathways, which are typical for the members of the *Ensifer*/*Sinorhizobium* clade [65,66,67,68,69]. The ‘*E. canadensis*’ SHC 2-14 possessed genes encoding 6-phospho-beta-glucosidase (EC: 3.2.1.86), fructose 1,6-bisphosphate 1-phosphatase (EC: 3.1.3.11), citrate (pro-3S)-lyase beta chain (EC: 4.1.3.6), gluconolactonase (EC: 3.1.1.17), and gluconate dehydratase (EC: 4.2.1.39) that the strain HO-A22^T^ did not possessed.

The mechanisms of reduction of nitrogen compounds by members of the *Ensifer*/*Sinorhizobium* group have been most fully studied using the *E*. (*S*.) *meliloti* strain by Torres et al. [70,71]. Strains HO-A22^T^ and ‘*E. canadensis*’ SHC 2-14 similarly possessed the *napADEF*, *nirKV*, and *norDEQ* genes, i.e., they were able to reduce nitrate to nitrous oxide due to the presence of ferredoxin-type periplasmic nitrate reductase (EC: 1.7.99.4), copper-containing nitrite reductase (NO-forming) (EC: 1.7.2.1), and nitric oxide reductase (EC: 1.7.99.7). At the same time, the genomes of both strains contained the *nirD* gene encoding nitrite reductase [NAD(P)H] (EC: 1.7.1.4). As a result, they could potentially carry out dissimilatory nitrite reduction to ammonium (DNRA pathway) as well. The ‘*E. canadensis*’ SHC 2-14 possesses the *nosDFRZ* genes encoding nitrous-oxide reductase (EC: 1.7.99.6); this corroborates the results of physiological experiments where the strain released molecular nitrogen into the gas phase during its growth [21]. It is important to highlight that the genome of strain SHC 2-14 is characterized by the absence of *nifH*, *nifX*, and *nifE* genes, which are associated with nitrogen fixation. These specific genes were previously identified in the genome of ‘*E. canadensis*’ T173^T^ and appear to have been acquired through horizontal transfer from *Sinorhizobium medicae* [6]. The strain HO-A22^T^ lacks these genes, so the terminal product of its denitrification was nitrous oxide. Nevertheless, this strain could potentially be used in biotechnologies to suppress the growth of sulfate-reducing bacteria (and therefore oil field equipment corrosion) by releasing the nitrite ion, an intermediate product of denitrification, if it accumulates in the formation water of an oil field [54].

Members of the *Ensifer*/*Sinorhizobium* clade are known for their ability to form extracellular polysaccharides. The *eps* and *exo* gene clusters have been identified as playing an essential role in exopolysaccharide production, which is vital for symbiotic rhizobial bacteria, since polysaccharides are directly involved in root nodule occupancy [72]. Bacteria of the genus *Ensifer* are typically nonsymbiotic species, but they are also capable of releasing extracellular polysaccharides. The *epsF* and *exoFQZ* genes encoding glycosyltransferase responsible for exopolysaccharide biosynthesis and the exopolysaccharide production protein were found in the genomes of strains HO-A22^T^ and SHC 2-14, which were therefore able to form these compounds and increase the viscosity of the cultivation media. In addition, the studied strains possessed the genes *rhaIMS* and *rfbCD*, responsible for L-rhamnose isomerization (EC: 5.3.1.14) and mutarotation (EC: 5.1.3.32) [72] and dTDP-4-dehydrorhamnose reduction (EC: 1.1.1.133) and epimerization (EC: 5.1.3.13) [73,74], respectively. These processes are known to be the key stages in the synthesis of succinoglycan exopolysaccharides [9,75,76], and, therefore, it becomes possible to suggest that strains HO-A22^T^ and SHC 2-14 produced this type of extracellular polysaccharide. The release of such compounds by strain HO-A22^T^ into the formation water of an oil reservoir may be of practical interest for microbial enhanced oil recovery.

Members of the class *Alphaproteobacteria* are frequently found in oil-contaminated soils due to their resistance to high concentrations of nonpolar pollutants and the ability to use various oil components for their growth [77]. The genomes of the strains HO-A22^T^ and SHC 2-14 contained a number of genes encoding the enzymes for aerobic degradation of long-chain fatty acids, aliphatic hydrocarbons, benzene, benzoate (BenB—benzoate 1,2-dioxygenase (EC: 1.14.12.10)), phenol, catechol (CatA—catechol 1,2-dioxygenase (EC: 1.13.11.1)), toluene, styrene, and phenanthrene. Various degradation pathways for phenanthrene and other PAHs have been described for the *Ensifer*/*Sinorhizobium* group [78,79,80]. The wide range of hydrocarbon compounds that strain HO-A22^T^ could potentially consume attests to its adaptation to the environmental conditions in petroleum reservoirs and the potential of its application in MEOR biotechnologies by stimulating its growth directly in the oil field.

Bacterial strains isolated from radionuclide-contaminated groundwater were found to be tolerant to the high concentrations of heavy metals due to the presence of HMRGs (heavy-metal resistance genes) in their genomes [29,81,82]. The strains HO-A22^T^ and SHC 2-14 were characterized by an abundance of HMRGs associated with copper, cadmium, zinc, lead, cobalt, mercuric, and chromium detoxification. Genomes of the strains contained the *petE* gene encoding plastocyanin that affected copper concentration and, therefore, provided tolerance to the ion [83]. The strains possessed the *cutCE* genes responsible for copper homeostasis as well, and that was their presumable alternate mechanism of copper resistance using cytoplasmic copper-binding protein CutE [84,85]. Strains HO-A22^T^ and ‘*E. canadensis*’ SHC 2-14 contained genes of the CadA enzyme—copper-translocating P-type ATPase (EC: 3.6.3.4), which is also known for cadmium, zinc, and lead detoxification [86]. The *copG* gene in both genomes encoded a copper amine oxidase, which was responsible for copper resistance as well [87]. Both strains were potentially tolerant of the mercury ions due to two different mechanisms: MerA enzyme is a widespread mercuric ion reductase (EC: 1.16.1.1), which is highly expressed under mercury stress in a number of prokaryotic organisms [88,89,90]; and ZntA protein is a lead-, cadmium-, zinc-, and mercury-transporting ATPase, which is found in phylogenetically closely related bacteria of the *Ensifer*/*Sinorhizobium* clade as well [8,91]. Finally, tolerance of the isolated strains to the chromium ions was possible due to the presence of the *chrAF* genes responsible for chromate transport (ChrA is a chromate efflux transporter) in their genomes [92,93]. The abundance of HMRGs in genomes of the strains HO-A22^T^ and ‘*E. canadensis*’ SHC 2-14 allowed them to inhabit environments highly polluted with heavy metals, such as radionuclide-contaminated groundwater.

Thus, the genomes of strains HO-A22^T^ and ‘*E. canadensis*’ SHC 2-14 contained a number of gene cluster activity which was responsible for their experimentally observed physiological characteristics. The isolates possessed the genes for various pathways of carbon metabolism, denitrification and reduction of nitrate to ammonium (DNRA process), synthesis of exopolysaccharides, presumably extracellular succinoglycan-type polysaccharides, degradation of aliphatic and aromatic hydrocarbon components of oil, and detoxification of various heavy metals and/or radionuclides, which allowed the studied strains to grow in the environmental conditions atypical for members of the *Ensifer*/*Sinorhizobium* clade and confirmed that they were adapted to the natural habitats from which they were isolated.

### 3.5. Physiological Characterization

Physiology of the strains HO-A22^T^ and ‘*E. canadensis*’ SHC 2-14 was investigated using TEG medium, typically at 24 °C because of the low temperature of their natural habitats. The strain HO-A22^T^ grew at 0–3% (*w*/*v*) NaCl in the medium (optimum, 0–1.2% NaCl); and ‘*E. canadensis*’ SHC 2-14 tolerated 0–1.6% (*w*/*v*) NaCl with an optimal growth at 0–0.5% NaCl. The strain HO-A22^T^ grew at 15–33 °C (optimum, 22–28 °C); and ‘*E. canadensis*’ SHC 2-14 tolerated 9–33 °C with an optimum at 28–30 °C. HO-A22^T^ grew at pH 6.0–9.8 with an optimal growth at pH 6.6–8.0; and the strain SHC 2-14 tolerated pH 5.9–9.2 (optimum, pH 6.6–8.6). Growth profiles of strains HO-A22T and SHC 2-14 at various temperatures, NaCl concentrations, and pH are shown in Figure S1. Both strains were catalase-positive. Strain HO-A22^T^ could grow anaerobically reducing nitrite to nitrous oxide (N_2_O) [94]; ‘*E. canadensis*’ SHC 2-14 could denitrify as well, but the terminal product of the process was molecular nitrogen (N_2_). Strains HO-A22^T^ and SHC 2-14 were resistant to carbenicillin (100 μg), erythromycin (15 μg), and kanamycin (30 μg), but sensitive to gentamicin (10 μg). 

In the API^®^ ZYM tests with the strains HO-A22^T^, SHC 2-14, *E. adhaerens* A^T^, and *E. morelensis* Lc04^T^, all four studied strains were positive or weakly positive for alkaline phosphatase, esterase (C4), esterase lipase (C8), α-chymotrypsin, acid phosphatase, naphthol-AS-BI-phosphohydrolase, ß-galactosidase, α-glucosidase, and ß-glucosidase, but negative for lipase (C14), α-galactosidase, ß-glucuronidase, α-mannosidase, and α-fucosidase. Strains HO-A22^T^ and SHC 2-14 were both positive for N-acetyl-ß-glucosaminidase (which only *E. morelensis* Lc04^T^ was negative for) and negative for cystine arylamidase and trypsin; HO-A22^T^ was positive for leucine arylamidase and weakly positive for valine arylamidase, whereas ‘*E. canadensis*’ SHC 2-14 was negative for both of them, unique among the studied strains. Results presented in Table S1 show the similarities in enzyme activities of the studied strains of the genus *Ensifer*. A number of diagnostic features were not given in the description of the species ‘*E. canadensis*’, and the results of the study of the strain SHC 2-14 complement the phenotypic properties of this species.

According to the API^®^ 20E results, strains HO-A22^T^, ‘*E. canadensis*’ SHC 2-14, *E. adhaerens* A^T^, and *E. morelensis* Lc04^T^ were positive or weakly positive for ß-galactosidase, tryptophane deaminase, rhamnose and arabinose fermentation/oxidation, and NO_2_ production, but negative for arginine dihydrolase, ornithine decarboxylase, citrate utilization, H_2_S production, indole production, acetoin production (Voges Proskauer), gelatinase, mannitol, inositol, sorbitol, sucrose, and amygdalin fermentation/oxidation. Strains HO-A22^T^ and ‘*E. canadensis*’ SHC 2-14 were both negative for lysine decarboxylase and urease (only the strain *E. adhaerens* A^T^ possessed both of them). HO-A22^T^ was positive for glucose and weakly positive for melibiose fermentation/oxidation, whereas ‘*E. canadensis*’ SHC 2-14 was negative for them. However, significant physiological differences between the strains were not observed in these experiments (Table S2).

In the API^®^ 50CH tests, all four studied strains showed positive or weakly positive reactions of glycerol, erythritol, D-arabinose, L-arabinose, D-ribose, D-xylose, L-xylose, D-adonitol, D-galactose, D-glucose, D-fructose, D-mannose, L-rhamnose, inositol, D-mannitol, D-sorbitol, aesculin (Fe citrate), D-cellobiose, D-maltose, D-sucrose, D-trehalose, D-turanose, D-lyxose, D-tagatose, D-fucose, L-fucose, D-arabite, and L-arabite utilization, but negative reactions of methyl-βD-xylopyranoside, inulin, starch, glycogen, gluconate K, and 5-ketogluconate K consumption. Both strains HO-A22^T^ and ‘*E. canadensis*’ SHC 2-14 did not utilize methyl-ɑD-mannopyranoside (only *E. morelensis* Lc04^T^ weakly consumed it) and D-melicitose (only *E. adhaerens* A^T^ did that weakly). HO-A22^T^ showed positive or weakly positive reactions of utilization of L-sorbose, dulcitol, N-acetylglucosamine, amygdalin, arbutin, salicin, and 2-ketogluconate K, whereas ‘*E. canadensis*’ SHC 2-14 showed negative reactions for these substrates. In our study, the ‘*E. canadensis*’ SHC 2-14 did not consume methyl-ɑD-glucopyranoside, D-lactose, D-melibiose, D-raffinose, xylitol, and gentiobiose; most of these features were indicated as weakly positive in the species description [6]. Results presented in Table 2 and Table S3 show common characteristics of the studied strains and several properties differentiating these strains from other species of the genus *Ensifer*.

The growth of the strains HO-A22^T^ and ‘*E. canadensis*’ SHC 2-14 on crude oil was investigated. After 30 days of cultivation at 30 °C, a decrease in the surface and interfacial tension in the cultivation medium by 5–10 mN/m was registered, which indicated the production of surfactants by strains. A decrease in the proportion of short-chain *n*-alkanes in the structure of oil saturated hydrocarbons degraded by each strain was also demonstrated (Figures S2 and S3), which indicated the consumption of these compounds by the new isolates. That is, both strains HO-A22^T^ and ‘*E. canadensis*’ SHC 2-14 were able to utilize oil components, which testifies their ability to produce oil-displacing metabolites promising for the enhancement of oil recovery.

The habitat from which ‘*E. canadensis*’ SHC 2-14 was isolated was characterized by a high content of radionuclides, metals and metalloids. In experiments with the salts of uranium, technetium, iron, chromium, vanadium, selenium, and molybdenum it was shown that the strain reduced more than 98% of V(V) and Se(VI) added to the media in the presence of sodium salts of acetic and lactic acids under anaerobic conditions (Figure S4); high reduction rates of Fe(III), Cr(VI), and U(VI) were noted: 87, 69, and 59% of the amount added into the medium, respectively. The content of Tc(VII) and Mo(VI) in the media decreased slightly. In the control experiment with cells killed by sterilization, there was no change in the concentration of the considered elements in the medium. Reduced radionuclides and heavy metals form extremely poorly soluble compounds and are not transported with groundwater, that is, the obtained results indicated the potential for the biogenic reduction of radionuclides, heavy metals and metalloids in groundwater in the zone of liquid radioactive waste disposal and the distribution of these elements in natural systems.

### 3.6. Chemotaxonomic Characterization

Cellular fatty acid profile of the strain HO-A22^T^ included C_18:1_ (66.8%), C_16:0_ (11.1%), C_14:0_ 3-OH (7.5%), C_16:1_ (4.8%), C_17:1_ (4.6%), C_18:0_ (3.3%), C_18:0_ 3-OH (1.1%), C_17:0_ (0.4%), and others (0.4%). Cellular fatty acid profile of the ‘*E. canadensis*’ SHC 2-14 included C_18:1_ (76.1%), C_16:0_ (10.5%), C_16:1_ (7.2%), C_14:0_ 3-OH (3.6%), C_18:0_ (2.4%), and C_17:0_ (0.2%). These profiles were compared with the those of the strains *E. adhaerens* A^T^ and *E. morelensis* Lc04^T^ (Table S4). C_18:1_ was the predominant compound in all studied profiles. Other dominant fatty acids were C_16:0_, C_16:1_, C_18:0_, and C_14:0_ 3-OH in all studies strains, except the strain *E. adhaerens* A^T^ containing the C_16:0_ 3-OH fatty acid instead of the C_14:0_ 3-OH. The fatty acid profile of the strain HO-A22^T^ was close to the one of *E. morelensis* Lc04^T^; the strain ‘*E. canadensis*’ SHC 2-14 lacked the widespread C_18:0_ 3-OH and C_17:1_ components. Although the strain ‘*E. canadensis*’ T173^T^ [6] was grown in a TY medium different from the TEG medium in which the four studied strains were grown, the major fatty acids C_18:1_, C_16:0_, C_18:0_, and C_16:1_ were common to the strain T173^T^ and reference strains of *Ensifer* species. 

The major respiratory quinone for the strains HO-A22^T^ and SHC 2-14 was Q10. The strain HO-A22^T^ did not contain any traces of other menaquinones, whereas the strain SHC 2-14 possessed the minor quinone Q9 (Figure S5). Two studied type strains of the genus *Ensifer*, as well as the strains HO-A22^T^ and SHC 2-14, contained phosphatidylcholines, phosphatidylethanolamines, phosphatidylglycerols, and diphosphatidylglycerols as major polar lipids of their cell membranes (Figures S6–S10). The ‘*E. canadensis*’ SHC 2-14 contained the unique polar lipid for this phylogenetic group, labeled as APL4, unidentified aminophospholipid.

Thus, four studied strains of the genus *Ensifer* had similar chemotaxonomic characteristics. All validly published members of the genus *Ensifer* were Gram-negative, motile, aerobic, non-spore-forming rods. They could reduce nitrate to nitrite, grow at 1% NaCl, 28 °C, pH 7.0–9.0, produce acid (API^®^ 50CH) from D-arabinose, D-fructose, D-glucose, D-maltose, D-mannose, D-ribose, D-trehalose, D-xylose, and erythritol; none of them grew at 4% NaCl, produced neither H_2_S nor acid from inulin or starch. Major fatty acids of the type strains were C_18:1_, C_16:0_, C_16:1_, and C_18:0_. Major polar lipids were phosphatidylcholines, phosphatidylethanolamines, and phosphatidylglycerols. Significant biochemical differences between the strain HO-A22^T^ and validly described species of the genus *Ensifer* were not observed; and ‘*E. canadensis*’ SHC 2-14 had slight peculiarities in its cellular fatty acid profile, respiratory quinones, and membranes polar lipids. These results confirmed that these properties of microorganisms have low taxonomic value.

## 4. Conclusions

Two novel strains, HO-A22^T^ and SHC 2-14, of the genus *Ensifer* isolated from water injected to the oil field and from radionuclide-contaminated groundwater, were analyzed using polyphasic taxonomy approaches and genomic analysis and compared with respective features of members of the genus *Ensifer*. Based on the phylogenetic, phenotypic, and chemotaxonomic analyses, it is concluded that strain HO-A22^T^ should be considered as a novel species of the genus *Ensifer*, for which the name *Ensifer oleiphilus* sp. nov. is proposed. The protologue of the proposed new species is presented in Table 3. The influence of environmental conditions such as salinity, pH, temperature, the content of crude oil or nitrates, heavy metals and radionuclides on the growth of strains revealed their adaptation to the environmental conditions. The strains HO-A22^T^ and SHC 2-14 can potentially be used in biotechnologies of microbial enhanced oil recovery, suppression of sulfide accumulation in oil reservoirs, and radionuclide pollution treatment due to their abilities to utilize hydrocarbons of crude oil, to produce extracellular polysaccharides, and to reduce nitrates and heavy metals. 

## Figures and Tables

**Table 1:** Comparison of the similarity level (%) of 16S rRNA genes and genomic indexes between the strains HO-A22^T^ and SHC 2-14 and type strains of the genus *Ensifer*.

Genome	HO-A22^T^					SHC 2-14				
	16S rRNA	dDDH	ANI	AAI	POCP	16S rRNA	dDDH	ANI	AAI	POCP
HO-A22^T^	100	100	100	100	100	99.8	35.8	88.7	91.6	80.6
SHC 2-14	99.8	35.8	88.7	91.7	80.6	100	100	100	100	100
*E. adhaerens* A^T^	99.1	25.4	84.1	84.1	79.7	99.2	25.4	83.8	83.5	78.9
*E. canadensis* T173^T^	99.8	35.8	87.9	91.8	80.8	100	92.8	99.1	99.1	88.7
*E. morelensis* Lc04^T^	99.9	45.9	92.4	95.1	87.4	99.9	35.6	88.7	91.4	79.3
*E. sesbaniae* CCBAU 65729^T^	99.0	25.6	83.7	84.3	75.6	99.1	25.8	83.7	83.8	74.1

ANI analysis of HO-A22^T^ genome was performed with the genomes of *Ensifer* strains. ANI values for the strain HO-A22^T^ were within the range of 83.7–92.4%, which were below the species threshold, proposed to be 95% [45,49]. The dDDH values against the reference genomes of *Ensifer* strains were within the range of 25.4–45.9% and were below the 70% threshold to differentiate bacterial species [45,47]. Thus, analysis of genome of the strain HO-A22^T^ testifies to its belonging to a novel species. The ANI and dDDH values between SHC 2-14 and ‘*E. canadensis*’ T173^T^ were 99.1% and 92.8%, respectively. These high values indicate that strain SHC 2-14 belongs to the ‘*E. canadensis*’ species.

**Table 2:** Characteristics differentiating strains HO-A22^T^ and ‘*E. canadensis*’ SHC 2-14 from members of the genus *Ensifer*.

Characteristics		‘*E. canadensis*’	‘*E. canadensis*’	*E. adhaerens*	*E. morelensis*	*E. sesbaniae*
Strain	HO-A22^T^	SHC 2-14	T173^T^	A^T^	Lc04^T^	CCBAU 65729^T^
Catalase	+	+	ND	w	+	–
Citrate utilization	–	–	ND	–	–	+
Urease	–	–	ND	+	–	+
Growth at:						
2% NaCl	+	–	+	+	+	–
3% NaCl	w	–	–	w	w	–
15 °C	+	+	+	+	+	–
37 °C	–	–	w	w	w	+
pH 5	–	–	+	–	–	–
pH 10	w	–	+	w	w	–
Acid production (API^®^ 50CH) from:						
D-fucose	+	+	–	+	+	ND
D-galactose	+	w	–	+	+	ND
D-lactose	+	–	w	+	+	+
D-melicitose	–	–	ND	w	–	+
D-turanose	+	w	–	+	+	ND
Dulcitol	+	–	ND	–	w	–
Gentiobiose	+	–	w	w	+	ND
L-rhamnose	+	w	–	+	+	+
L-sorbose	+	–	ND	–	w	+
Salicin	+	–	–	–	+	+
Major fatty acids	C_18:1_, C_16:0_, C_14:0_ 3-OH, C_16:1_, C_17:1_	C_18:1_, C_16:0_, C_16:1_, C_14:0_ 3-OH, C_18:0_	C_18:1_, C_16:0_, C_12:0_ aldehyde, C_18:0_, C_18:0_ cyclo ω8c	C_18:1_, C_16:0_, C_16:1_, C_14:0_ 3-OH, C_18:0_, C_18:0_ 3-OH	C_18:1_, C_16:0_, C_16:0_ 3-OH, C_18:0_, C_16:1_	C_18:1_, C_12:0_ aldehyde, C_16:0_, C_16:1_, C_18:0_
Major polar lipids	PC, PE, DPG, PG	PC, DPG, PE, APL4, PG	ND	PE, PC, PG, DPG	PE, DPG, PC, PG	PC, PE, PG
G+C content, %	61.7	61.0	61.0	62.8 ^a^	61.7 ^b^	60.4
Isolation source	Injection water of an oil field	Radionuclide-contaminated groundwater	Root nodule of *Melilotus albus*	Soil ^a^	Root nodule of *Leucaena leucocephala* ^b^	Root nodule of *Sesbania cannabina*

All strains were Gram-negative, motile, aerobic, non-spore-forming rods. Positive results for all strains were obtained for: growth at pH 7.0–9.0, 28 °C, 1% NaCl; acid production (API^®^ 50CH) from D-fructose, D-glucose, D-mannose, and D-trehalose. Negative results for all strains were obtained for growth at 4% NaCl. Data for ‘*E. canadensis*’ T173^T^ are from Bromfield et al. [6], and those of *E. sesbaniae* CCBAU 65729^T^ are from Wang et al. [4]. Data for all other strains are from this study, except as labeled: ^a^, Data from Tóth et al. [95]; ^b^, Data from Wang et al. [96]. Designations: +, positive; –, negative; w, weakly positive; APL4, unidentified aminophospholipid; DPG, diphosphatidylglycerols; PC, phosphatidylcholines; PE, phosphatidylethanolamines; PG, phosphatidylglycerols; ND, not determined.

**Table 3:** Protologue description of *Ensifer oleiphilus* sp. nov.

Parameter	*Ensifer oleiphilus* sp. nov.
Genus name	*Ensifer*
Species name	*Ensifer oleiphilus*
Species status	sp. nov.
Species etymology	o.le.i.phi’lus. L. neut. n. *oleum* oil; N.L. masc. adj. *philus* (from Gr. masc. adj. *philos*) loving; N.L. masc. adj. *oleiphilus* loving oil, referring to the ability of type strain to utilize hydrocarbons of crude oil
Description of the new taxon and diagnostic traits	Cells are Gram-negative, motile, aerobic, non-spore-forming rods. Colonies up to 8.0 in diameter are creamy, smooth, shiny, and round after 5 days of cultivation in the TEG medium at 22 °C. Growth occurs on TEG, PCA, and LB media, in the presence of 0–3.0% (*w*/*v*) NaCl (optimum, 0–1.2% NaCl), at pH 6.0–9.8 (optimum, pH 6.6–8.0) and at 15–33 °C (optimum, 22–28 °C). Catalase-positive, but negative for urease and citrate utilization. Chemoorganoheterotrophic, facultatively anaerobic. Nitrate is reduced to the nitrous oxide. Acid is produced from D-fucose, D-galactose, D-lactose, D-turanose, dulcitol, gentiobiose, L-rhamnose, L-sorbose, and salicin, but not from D-melicitose. The predominant fatty acids are C_18:1_, C_16:0_, C_14:0_ 3-OH, C_16:1_, C_17:1_, and C_18:0_. Major menaquinone is Q10. The major polar lipids are phosphatidylcholine, phosphatidylethanolamine, diphosphatidylglycerol, and phosphatidylglycerol. The genome size of the type strain is 6.76 Mb with a G+C value of 61.7%. The strain type, HO-A22^T^ (=VKM B-3646^T^ = KCTC 92427^T^), was isolated from a denitrifying enrichment obtained from an injection water of the Cheremukhovskoe oil field (Tatarstan, Russia). The GenBank/EMBL/DDBJ accession number for the 16S rRNA gene sequence is MT495799 and the genomic assembly accession number is GCF_013371465.1.
Country of origin	Russian Federation
Region of origin	Nurlat region, Tatarstan
Date of isolation	2018
Source of isolation	Injection water of an oil field
Sampling date	2016
Latitude	55°0′ N
Longitude	51°1′ E
Depth (meters below sea level)	850–1300
Number of strains in study	1
Information related to the Nagoya Protocol	Not applicable

## Data Availability

The 16S rRNA gene sequences of strains HO-A22^T^ and SHC 2-14 have been deposited at the GenBank/EMBL/DDBJ under accession numbers MT495799 and MG051317, respectively. The genomic assemblies of strains HO-A22^T^ and SHC 2-14 have been deposited at the Gen-Bank/EMBL/DDBJ under accession numbers GCF_013371465.1 (JABWDU000000000.1) and GCF_013141885.1 (SROZ00000000.1), respectively.

## Supplementary Materials

The following supporting information can be downloaded at: https://www.mdpi.com/article/10.3390/microorganisms11092314/s1, Figure S1: Growth profiles of strains HO-A22^T^ (a–c) and ‘*E. canadensis*’ SHC 2-14 (d–f) in the TEG medium at various temperatures (a,d), NaCl concentrations (*w*/*v*, %) (b,e), and pH (c,f); Figure S2: Chromatograms of the saturated hydrocarbon fraction of sterile oil (a) and oil degraded by the strain HO-A22^T^ (b) and residual content of alkanes (%) in oil degraded by the strain HO-A22^T^ (c). The strain was incubated in a liquid medium with crude oil as a substrate at 30 °C for 30 days; Figure S3: Chromatograms of the saturated hydrocarbon fraction of sterile oil (a) and oil degraded by the strain ‘*E. canadensis*’ SHC 2-14 (b) and residual content of alkanes (%) in oil degraded by the strain SHC 2-14 (c). The strain was incubated in a liquid medium with crude oil as a substrate at 30 °C for 30 days; Figure S4: Residual content (%) of Se(VI), V(V), Fe(III), Cr(VI), U(VI), Tc(VII), and Mo(VI) in the medium after incubation of the strain ‘*E. canadensis*’ SHC 2-14 at 22 °C for 10 days. The strain grew in the Adkins mineral medium with sodium acetate (20 mM) or sodium lactate (30 mM); Figure S5: Mass spectra of menaquinones from the strains HO-A22^T^ (a,b) and ‘*E. canadensis*’ SHC 2-14 (c,d), showing the presence of menaquinone Q10; Figure S6: Two-dimensional thin layer chromatogram of polar lipids from strains HO-A22^T^ (a), ‘*E. canadensis*’ SHC 2-14 (b), *E. adhaerens* A^T^ (c), and *E. morelensis* Lc04^T^ (d). The components were visualized by staining with 5% sulfuric acid in ethanol and heating at 180 °C for 15 min. Abbreviations: PC, phosphatidylcholines; PE, phosphatidylethanolamines; DPG, diphosphatidylglycerols; PG, phosphatidylglycerols; PME, phosphatidylmethylethanolamines; PS, phosphatidylserines; APL1–APL5, aminophospholipids; PL1–PL5, phospholipids; GL1–GL3, glycolipids; GPL1, glycophospholipid; AL1, aminolipid; PA, phosphatidic acids; LPE, lysophosphatidylethanolamines; X, unidentified lipid; Figure S7: Identification of polar lipids from the strain HO-A22^T^. The components were visualized by molybdenum blue (a); α-naphthol (b); ninhydrin (c); and Dragendorff (d). Abbreviations: AL, aminolipids; GL, glycolipids; PC, phosphatidylcholines; PE, phosphatidylethanolamines; PL, phospholipids; Figure S8: Identification of polar lipids from the strain ‘*E. canadensis*’ SHC 2-14. The components were visualized by molybdenum blue (a); α-naphthol (b); ninhydrin (c); and Dragendorff (d). Abbreviations: AL, aminolipids; PC, phosphatidylcholines; PL, phospholipids; Figure S9: Identification of polar lipids from the strain *E. adhaerens* A^T^. The components were visualized by molybdenum blue (a); α-naphthol (b); ninhydrin (c); and Dragendorff (d). Abbreviations: AL, aminolipids; DPG, diphosphatidylglycerols; GL, glycolipids; LPE, lysophosphatidylethanolamines; PA, phosphatidic acids; PC, phosphatidylcholines; PE, phosphatidylethanolamines; PG, phosphatidylglycerols; PL, phospholipids; PS, phosphatidylserines; Figure S10: Identification of polar lipids from the strain *E. morelensis* Lc04^T^. The components were visualized by molybdenum blue (a); α-naphthol (b); ninhydrin (c); and Dragendorff (d). Abbreviations: AL, aminolipids; DPG, diphosphatidylglycerols; LPE, lysophosphatidylethanolamines; PA, phosphatidic acids; PC, phosphatidylcholines; PE, phosphatidylethanolamines; PG, phosphatidylglycerols; PL, phospholipids; PS, phosphatidylserines; Table S1: Enzymatic activities of the strains HO-A22^T^, ‘*E. canadensis*’ SHC 2-14, *E. adhaerens* A^T^, and *E. morelensis* Lc04^T^ determined by the API^®^ ZYM test (bioMérieux, France). Designations: +, positive; –, negative; w, weakly positive; Table S2: Comparison of enzymatic activities of the strains HO-A22^T^, ‘*E. canadensis*’ SHC 2-14, *E. adhaerens* A^T^, and *E. morelensis* Lc04^T^, determined by the API^®^ 20E test (bioMérieux, France). Designations: +, positive; –, negative; w, weakly positive; Table S3: Carbohydrate fermentation by the strains HO-A22^T^, ‘*E. canadensis*’ SHC 2-14, *E. adhaerens* A^T^, and *E. morelensis* Lc04^T^, determined by the API^®^ 50CH test (bioMérieux, France). Designations: +, positive; –, negative; w, weakly positive; Table S4: Cellular fatty acid profiles of the strains HO-A22^T^, ‘*E. canadensis*’ SHC 2-14, *E. adhaerens* A^T^, and *E. morelensis* Lc04^T^.
