# Supplementary material for: Genome Analysis and Potential Ecological Functions of Members of the Genus Ensifer from Subsurface Environments and Description of Ensifer oleiphilus sp. nov
Source: Microorganisms. 2023 Sep 14;11(9):2314. doi: 10.3390/microorganisms11092314 (PMC10538136; doi:10.3390/microorganisms11092314)
Supplement: Supplementary file 1 [file microorganisms-11-02314-s001.zip › microorganisms-2548101-supplementary.pdf]

microorganisms

MDPI

Supplementary Materials

Genome Analysis and Potential Ecological Functions of Members of the Genus *Ensifer* from Subsurface Environments and Description of *Ensifer oleiphilus* sp. nov.

Alexey P. Ershov [1], Tamara L. Babich [1], Denis S. Grouzdev [2], Diyana S. Sokolova [1], Ekaterina M. Semenova [1], Alexander N. Avtukh [3], Andrey B. Poltaraus [4], Elena A. Ianutsevich [1] and Tamara N. Nazina [1,*]

[1] Winogradsky Institute of Microbiology, Research Center of Biotechnology of the Russian Academy of Sciences, 119071 Moscow, Russia; sokolovadiyana@gmail.com (D.S.S.); semenova_inmi@mail.ru (E.M.S.); e.a.ianutsevich@gmail.com (E.A.I.); nazina@inmi.ru (T.N.N.)
[2] SciBear OU, 10115 Tallinn, Estonia; denisgrouzdev@gmail.com (D.S.G.)
[3] Skryabin Institute of Biochemistry and Physiology of Microorganisms, Russian Academy of Sciences, Pushchino Scientific Center for Biological Research of the Russian Academy of Sciences, 142290 Pushchino, Russia; avtukh@rambler.ru (A.N.A.)
[4] Engelhardt Institute of Molecular Biology, Russian Academy of Sciences, 119991 Moscow, Russia; abpolt@gmail.com (A.B.P.)

* Correspondence: nazina@inmi.ru; Tel.: +7-499-135-0341

Supplementary Figures S1-S10
Supplementary Tables S1-S4

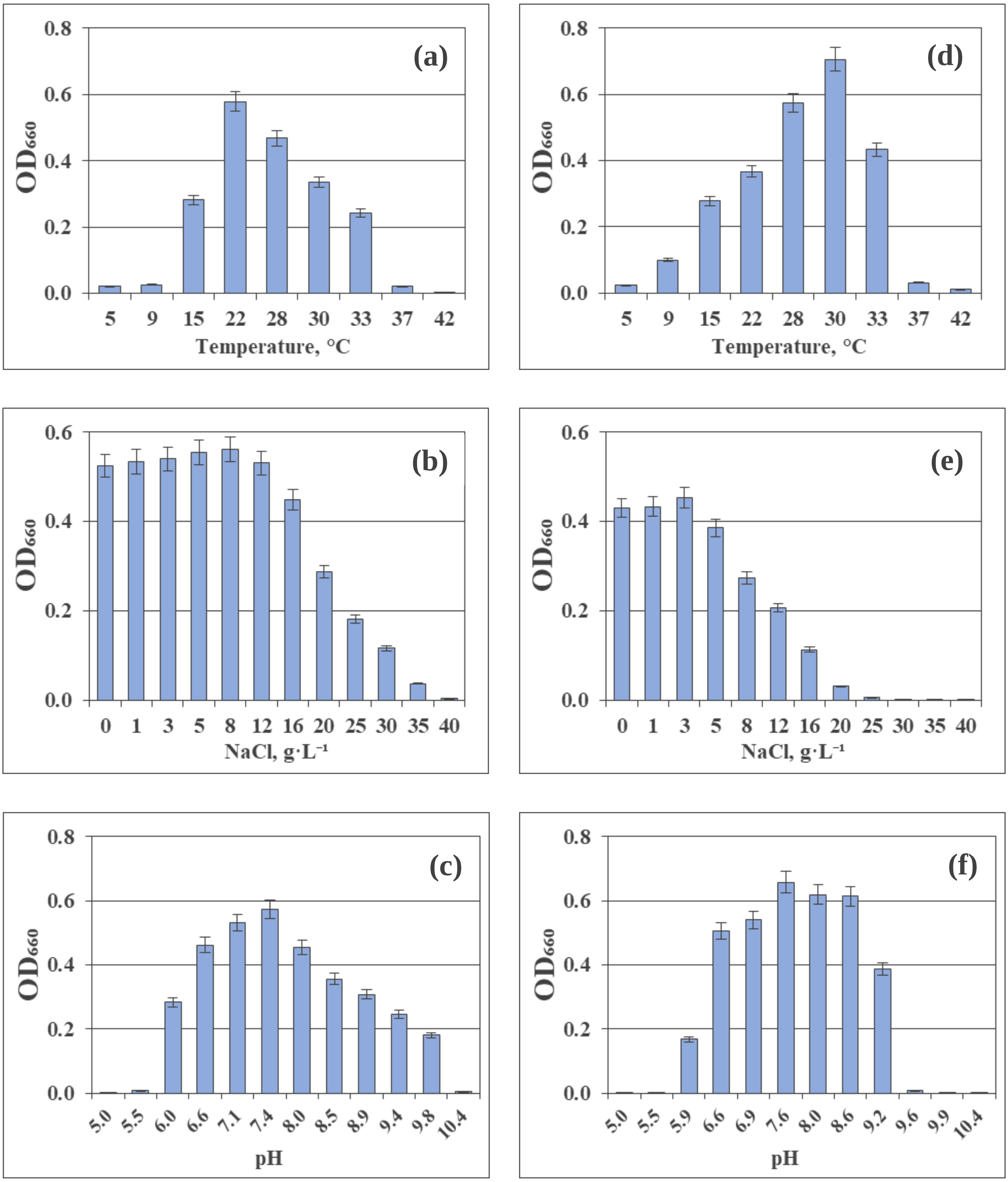

Figure S1. Growth profiles of strains HO-A22^T (a–c) and '*E. canadensis*' SHC 2-14 (d–f) in the TEG medium at various temperatures (a, d), NaCl concentrations (w/v, %) (b, e), and pH (c, f).

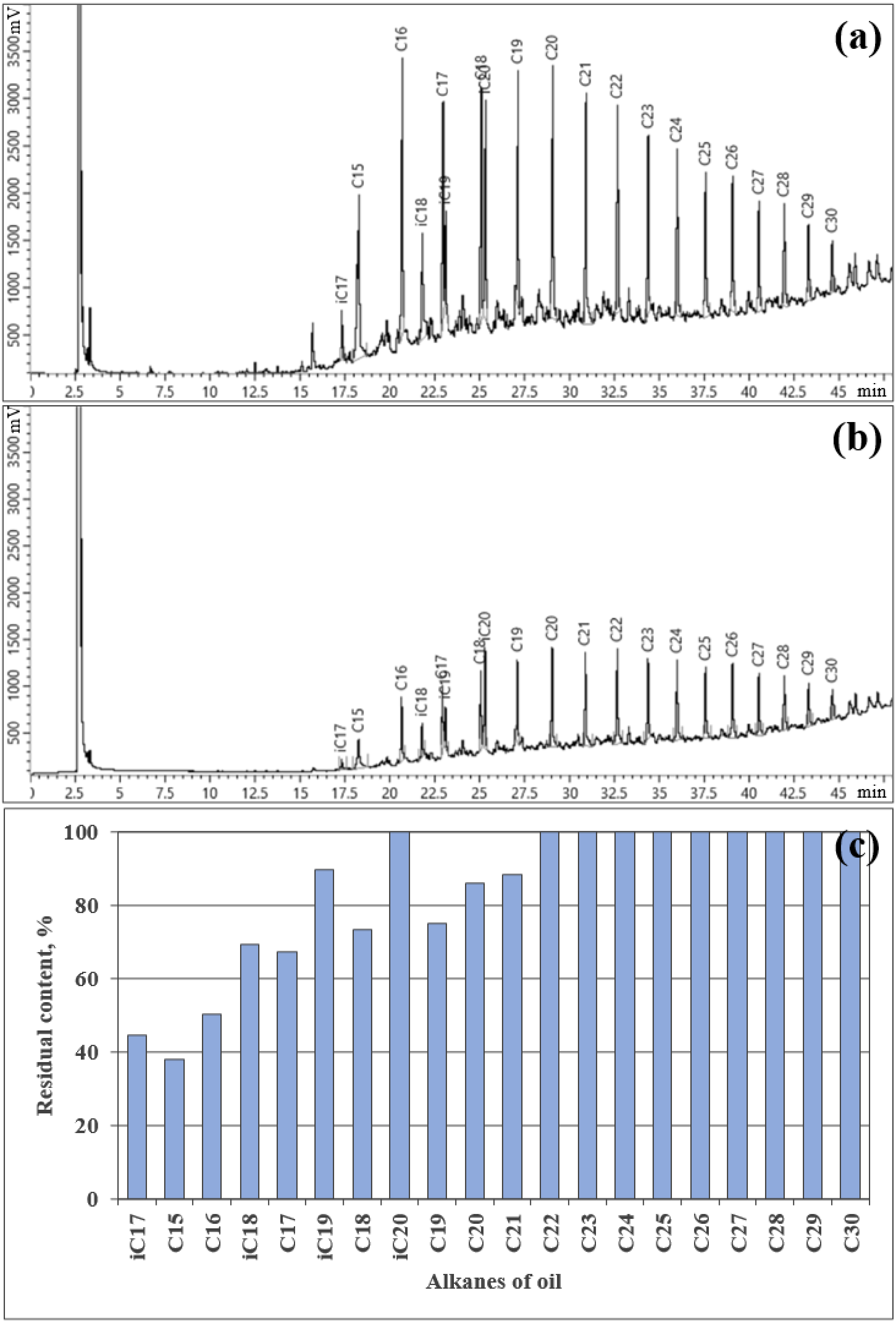

Figure S2. Chromatograms of the saturated hydrocarbon fraction of sterile oil (a) and oil degraded by the strain HO-A22^{T} (b) and residual content of alkanes (%) in oil degraded by the strain HO-A22^{T} (c). The strain was incubated in a liquid medium with crude oil as a substrate at 30 °C for 30 days.

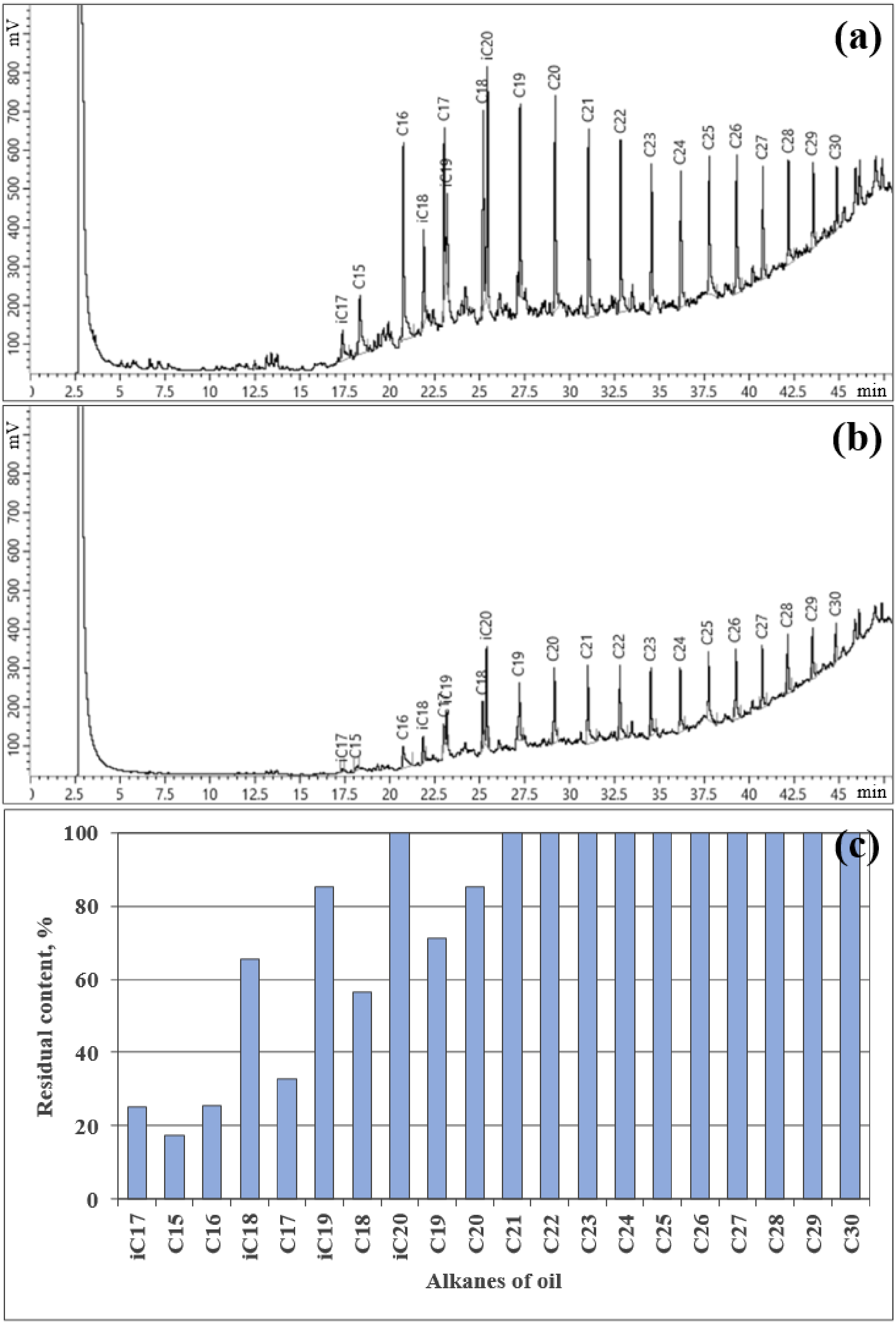

Figure S3. Chromatograms of the saturated hydrocarbon fraction of sterile oil (a) and oil degraded by the strain '*E. canadensis*' SHC 2-14 (b) and residual content of alkanes (%) in oil degraded by the strain SHC 2-14 (c). The strain was incubated in a liquid medium with crude oil as a substrate at 30 °C for 30 days.

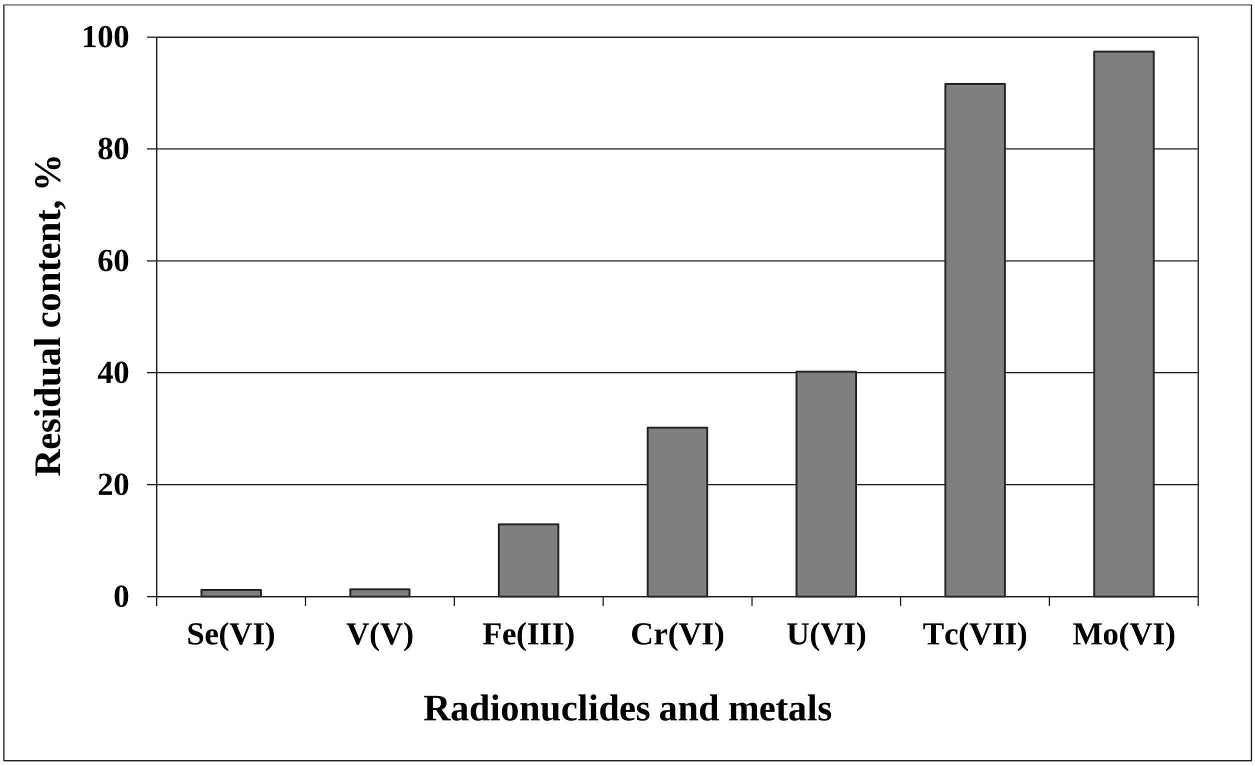

Figure S4. Residual content (%) of Se(VI), V(V), Fe(III), Cr(VI), U(VI), Tc(VII), and Mo(VI) in the medium after incubation of the strain '*E. canadensis*' SHC 2-14 at 22 °C for 10 days. The strain grew in the Adkins mineral medium with sodium acetate (20 mM) or sodium lactate (30 mM).

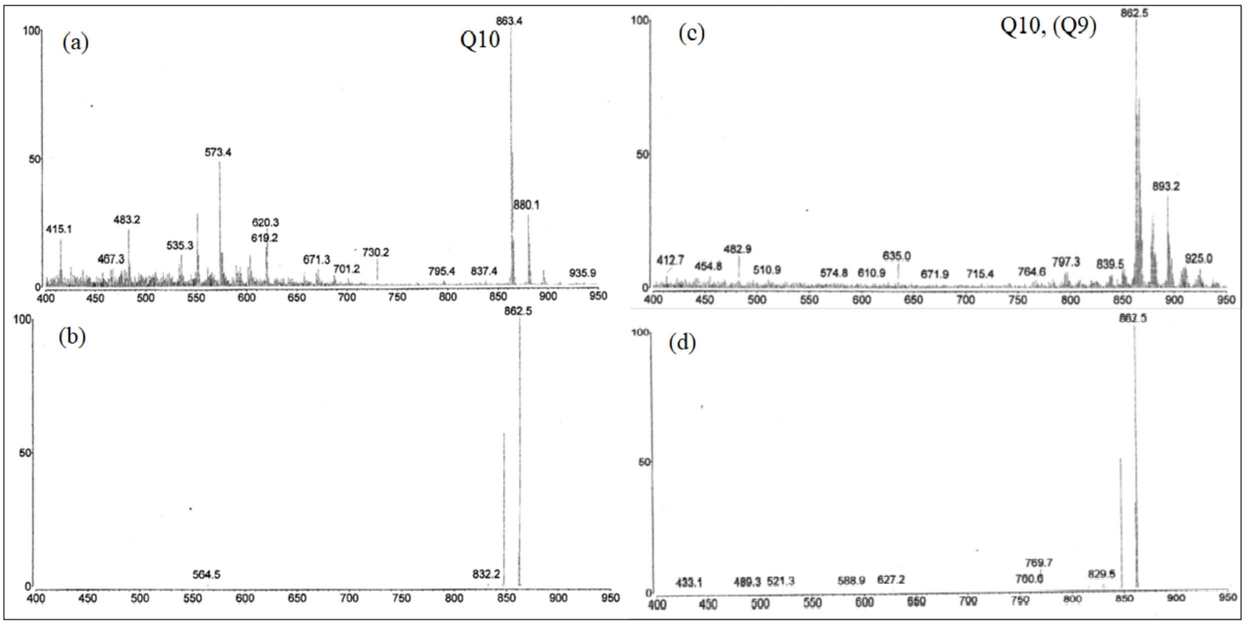

Figure S5. Mass spectra of menaquinones from the strains HO-A22^{T} (a, b) and '*E. canadensis*' SHC 2-14 (c, d), showing the presence of menaquinone Q10.

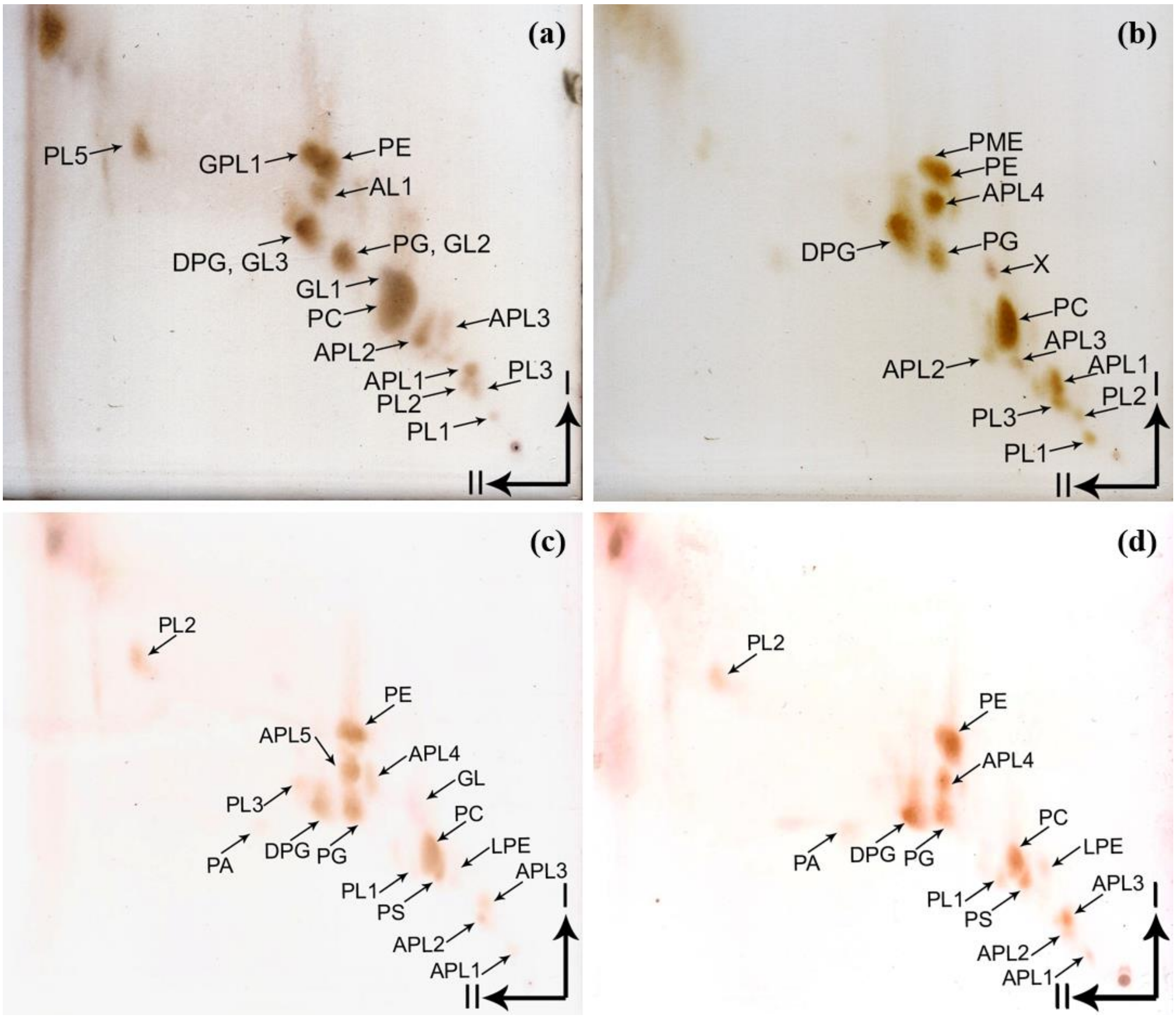

Figure S6. Two-dimensional thin layer chromatogram of polar lipids from strains HO-A22^T (a), '*E. canadensis*' SHC 2-14 (b), *E. adhaerens* A^T (c), and *E. morelensis* Lc04^T (d). The components were visualized by staining with 5% sulfuric acid in ethanol and heating at 180 °C for 15 min. Abbreviations: PC, phosphatidylcholines; PE, phosphatidylethanolamines; DPG, diphosphatidylglycerols; PG, phosphatidylglycerols; PME, phosphatidylmethylethanolamines; PS, phosphatidylserines; APL1–APL5, aminophospholipids; PL1–PL5, phospholipids; GL1–GL3, glycolipids; GPL1, glycophospholipid; AL1, aminolipid; PA, phosphatidic acids; LPE, lysophosphatidylethanolamines; X, unidentified lipid.

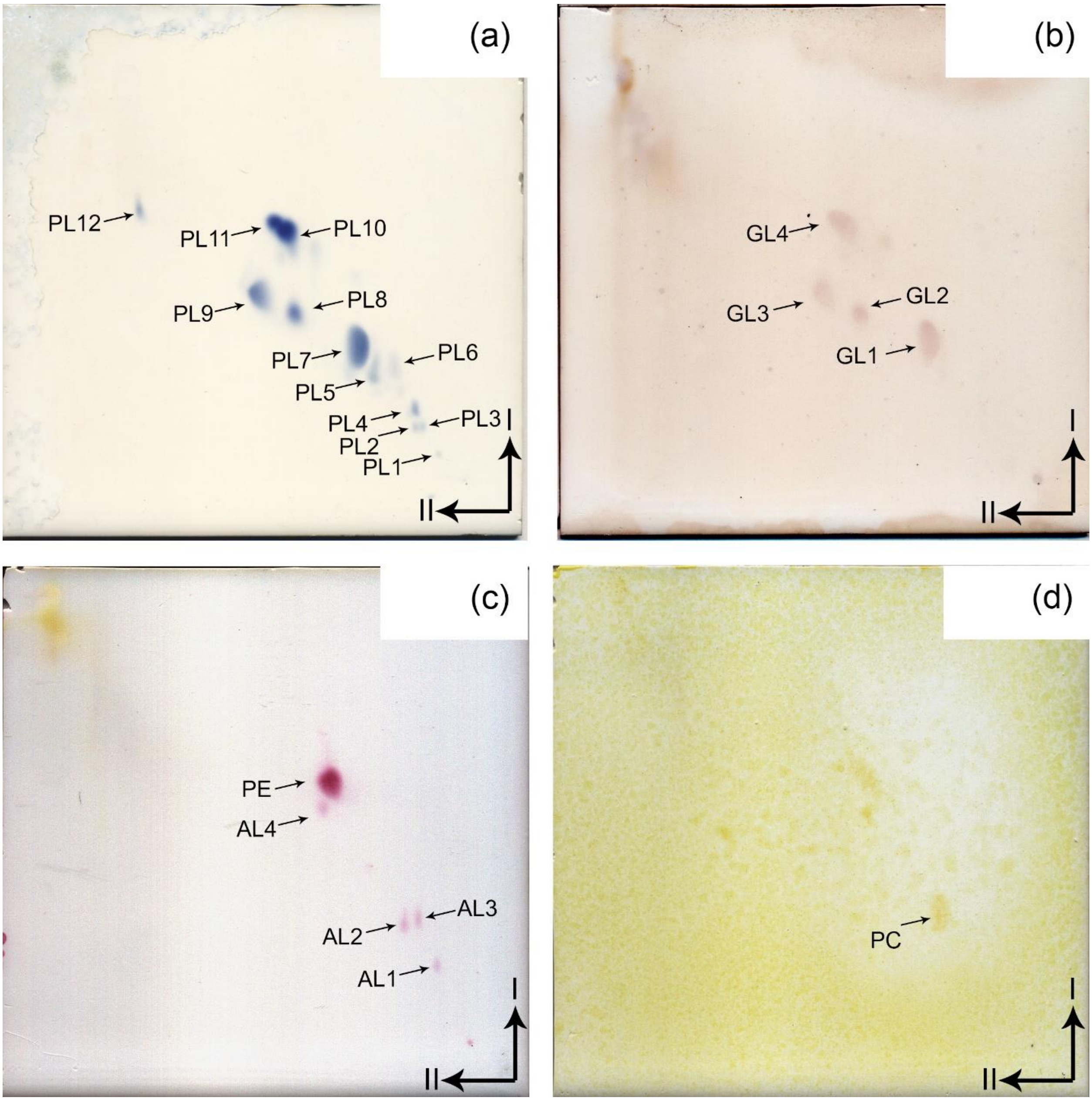

Figure S7. Identification of polar lipids from the strain HO-A22^{T}. The components were visualized by molybdenum blue (a); α-naphthol (b); ninhydrin (c); and Dragendorff (d). Abbreviations: AL, aminolipids; GL, glycolipids; PC, phosphatidylcholines; PE, phosphatidylethanolamines; PL, phospholipids.

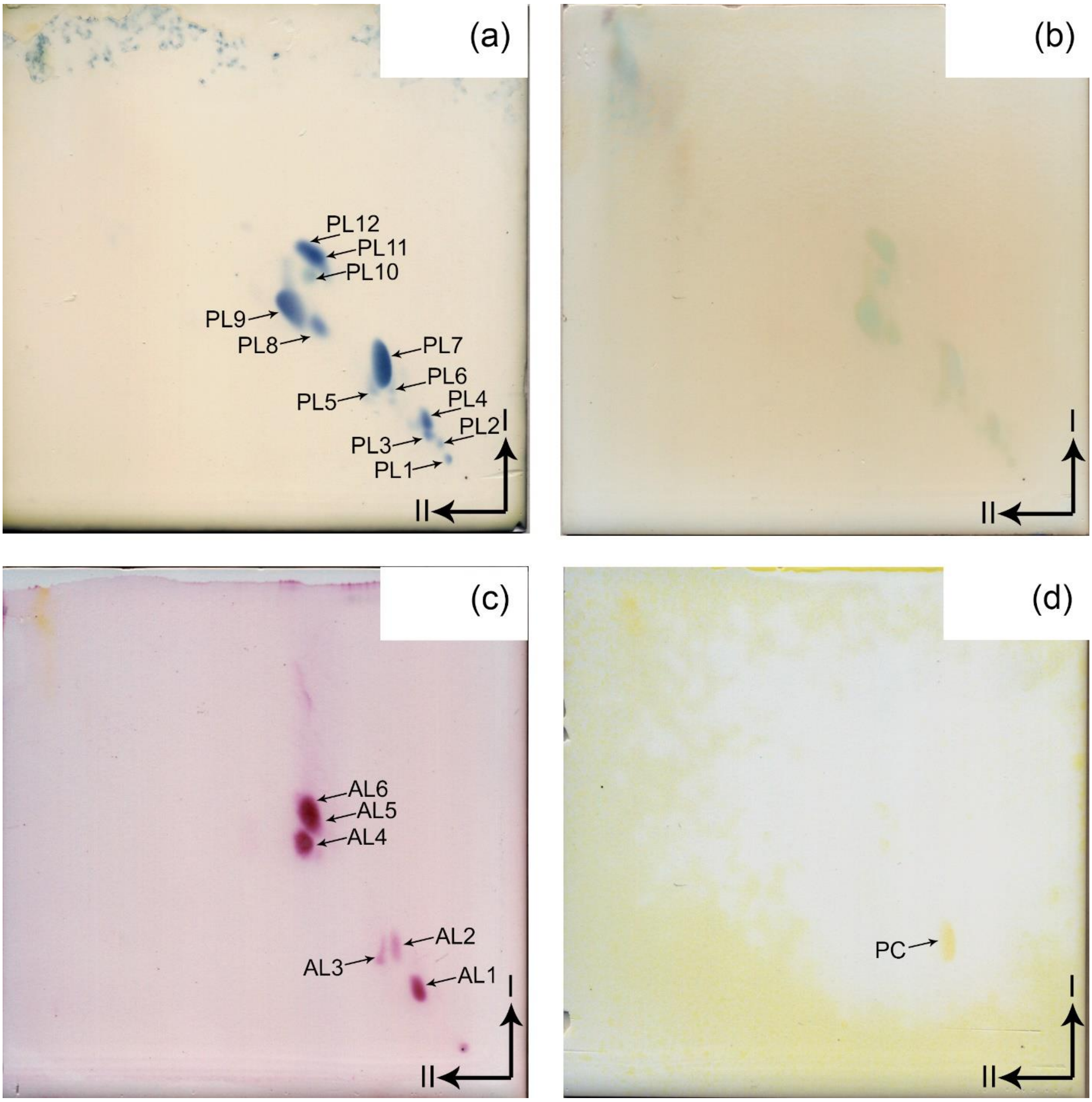

Figure S8. Identification of polar lipids from the strain '*E. canadensis*' SHC 2-14. The components were visualized by molybdenum blue (a); α-naphthol (b); ninhydrin (c); and Dragendorff (d). Abbreviations: AL, aminolipids; PC, phosphatidylcholines; PL, phospholipids.

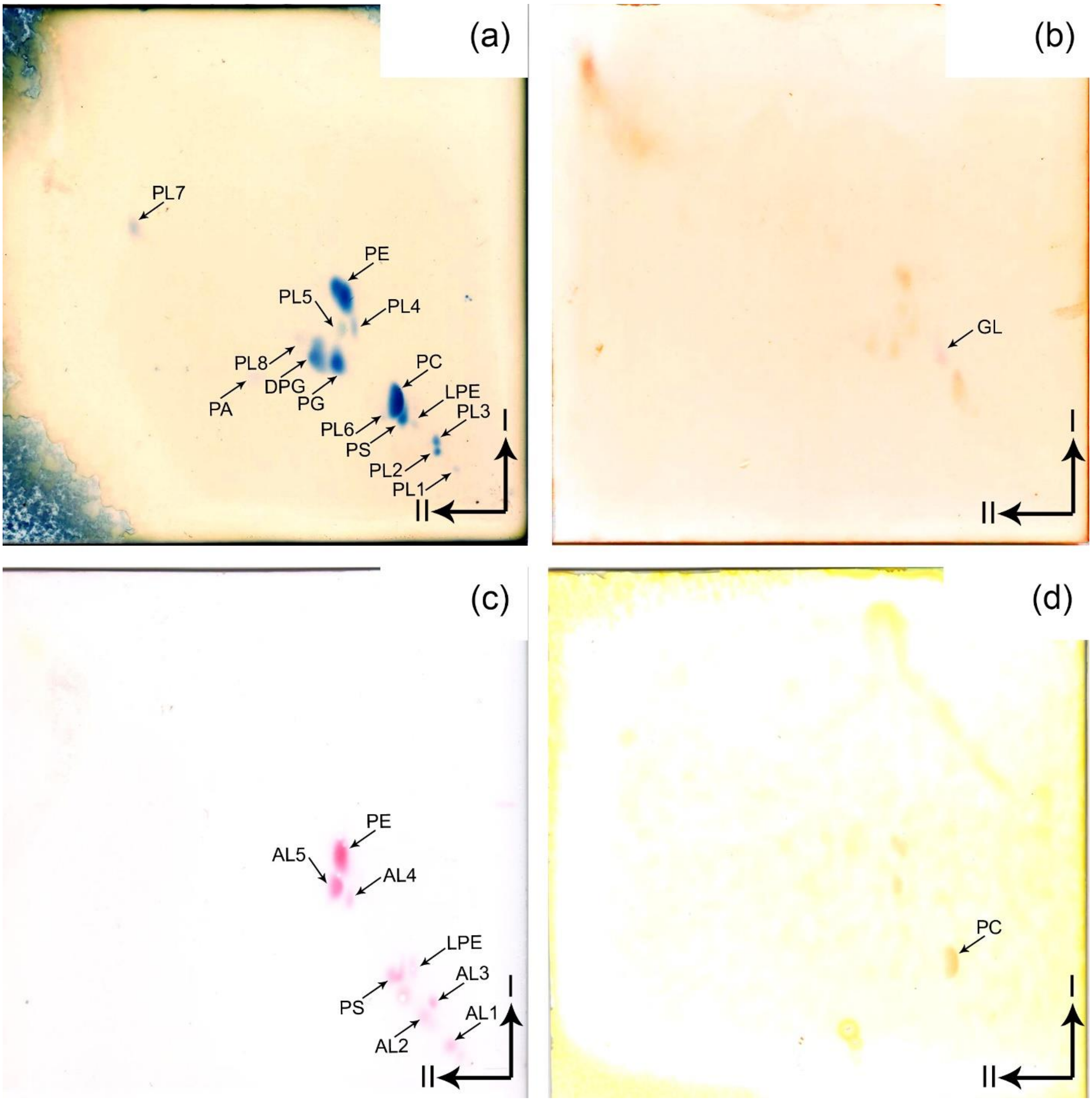

Figure S9. Identification of polar lipids from the strain *E. adhaerens* A^T. The components were visualized by molybdenum blue (a); α-naphthol (b); ninhydrin (c); and Dragendorff (d). Abbreviations: AL, aminolipids; DPG, diphosphatidylglycerols; GL, glycolipids; LPE, lysophosphatidylethanolamines; PA, phosphatidic acids; PC, phosphatidylcholines; PE, phosphatidylethanolamines; PG, phosphatidylglycerols; PL, phospholipids; PS, phosphatidylserines.

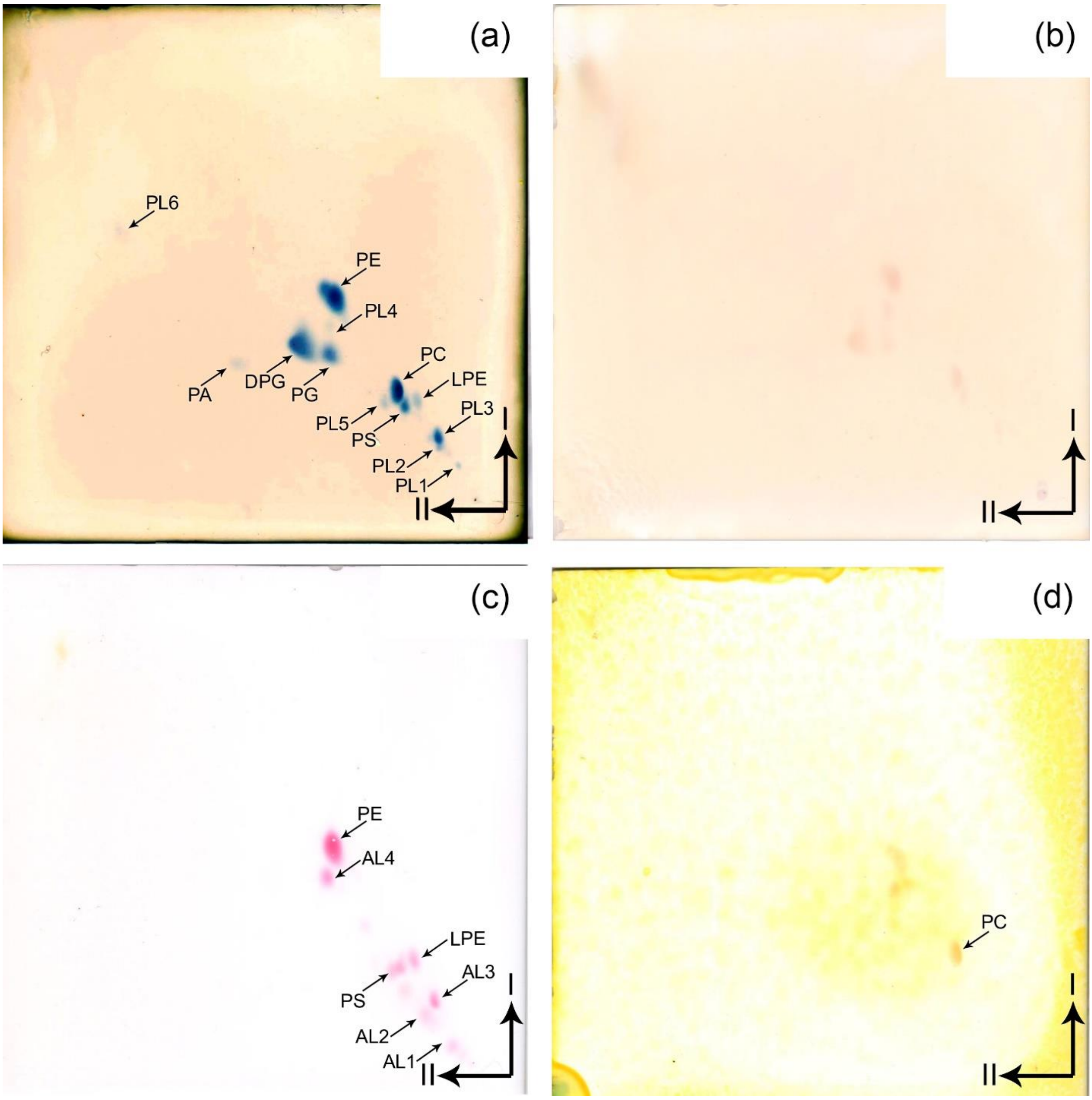

Figure S10. Identification of polar lipids from the strain *E. morelensis* Lc04ᵀ. The components were visualized by molybdenum blue (a); α-naphthol (b); ninhydrin (c); and Dragendorff (d). Abbreviations: AL, aminolipids; DPG, diphosphatidylglycerols; LPE, lysophosphatidylethanolamines; PA, phosphatidic acids; PC, phosphatidylcholines; PE, phosphatidylethanolamines; PG, phosphatidylglycerols; PL, phospholipids; PS, phosphatidylserines.

Table S1. Comparison of enzymatic activities of the strains HO-A22^T, '*E. canadensis*' SHC 2-14, *E. adhaerens* A^T, and *E. morelensis* Lc04^T, determined by the API® ZYM test (bioMérieux, France). Designations: +, positive; –, negative; w, weakly positive.

ENZYME	HO-A22^T	'*E. canadensis*' SHC 2-14	*E. adhaerens* A^T	*E. morelensis* Lc04^T
Alkaline phosphatase	+	w	+	w
Esterase (C4)	w	w	+	w
Esterase lipase (C8)	w	w	w	w
Lipase (C14)	–	–	–	–
Leucine arylamidase	+	–	+	+
Valine arylamidase	w	–	+	w
Cystine arylamidase	–	–	+	w
Trypsin	–	–	+	+
α-Chymotrypsin	w	w	+	+
Acid phosphatase	+	+	+	+
Naphthol-AS-BI-phosphohydrolase	+	+	+	+
α-Galactosidase	–	–	–	–
ß-Galactosidase	+	w	w	+
ß-Glucuronidase	–	–	–	–
α-Glucosidase	+	+	+	+
ß-Glucosidase	+	+	+	+
N-acetyl-ß-glucosaminidase	+	+	+	–
α-Mannosidase	–	–	–	–
α-Fucosidase	–	–	–	–

Table S2. Enzymatic activities of the strains HO-A22^T, '*E. canadensis*' SHC 2-14, *E. adhaerens* A^T, and *E. morelensis* Lc04^T determined by the API® 20E test (bioMérieux, France). Designations: +, positive; –, negative; w, weakly positive.

REACTIONS	HO-A22^T	'*E. canadensis*' SHC 2-14	*E. adhaerens* A^T	*E. morelensis* Lc04^T
ß-Galactosidase	w	w	w	w
Arginine dihydrolase	–	–	–	–
Lysine decarboxylase	–	–	+	–
Ornithine decarboxylase	–	–	–	–
Citrate utilization	–	–	–	–
H_2S production	–	–	–	–
Urease	–	–	+	–
Tryptophane deaminase	w	+	w	+
Indole production	–	–	–	–
Acetoin production (Voges Proskauer)	–	–	–	–
Gelatinase fermentation/oxidation	–	–	–	–
Glucose fermentation/oxidation	+	–	–	w
Mannitol fermentation/oxidation	–	–	–	–
Inositol fermentation/oxidation	–	–	–	–
Sorbitol fermentation/oxidation	–	–	–	–
Rhamnose fermentation/oxidation	+	w	+	w
Sucrose fermentation/oxidation	–	–	–	–
Melibiose fermentation/oxidation	w	–	w	–
Amygdalin fermentation/oxidation	–	–	–	–
Arabinose fermentation/oxidation	+	w	+	+
$NO_3^- \rightarrow NO_2^-$	+	+	w	+

Table S3. Carbohydrate fermentation by the strains HO-A22^T, '*E. canadensis*' SHC 2-14, *E. adhaerens* A^T, and *E. morelensis* Lc04^T, determined by the API® 50CH test (bioMérieux, France). Designations: +, positive; –, negative; w, weakly positive.

**SUBSTRATE**	**HO-A22^T**	***'E. canadensis'* SHC 2-14**	***E. adhaerens* A^T**	***E. morelensis* Lc04^T**
**1**	**2**	**3**	**4**	**5**
Glycerol	+	w	+	+
Erythritol	+	w	w	+
D-Arabinose	+	w	+	+
L-Arabinose	+	+	+	+
D-Ribose	+	+	+	+
D-Xylose	+	+	+	+
L-Xylose	+	w	+	+
D-Adonitol	+	w	+	+
Methyl-βD-xylopyranoside	–	–	–	–
D-Galactose	+	w	+	+
D-Glucose	+	w	+	+
D-Fructose	+	w	+	+
D-Mannose	+	w	+	+
L-Sorbose	+	–	–	w
L-Rhamnose	+	w	+	+
Dulcitol	+	–	–	w
Inositol	+	w	+	+
D-Mannitol	+	w	+	+
D-Sorbitol	+	w	+	+
Methyl-αD-mannopyranoside	–	–	–	w
Methyl-αD-glucopyranoside	+	–	w	+
N-acetylglucosamine	+	–	–	+
Amygdalin	+	–	–	w

Supplementary Table S3 (continuation)

1	2	3	4	5
Arbutin	+	–	–	w
Aesculin (Fe citrate)	+	+	+	+
Salicin	+	–	–	+
D-Cellobiose	+	w	+	+
D-Maltose	+	w	+	+
D-Lactose	+	–	+	+
D-Melibiose	w	–	+	+
D-Sucrose	+	w	+	+
D-Trehalose	+	w	+	+
Inulin	–	–	–	–
D-Melicitose	–	–	w	–
D-Raffinose	w	–	w	w
Starch	–	–	–	–
Glycogen	–	–	–	–
Xylitol	+	–	+	+
Gentiobiose	+	–	w	+
D-Turanose	+	w	+	+
D-Lyxose	+	w	+	+
D-Tagatose	+	+	+	+
D-Fucose	+	+	+	+
L-Fucose	+	+	+	+
D-Arabite	+	w	+	+
L-Arabite	+	w	w	+
Gluconate K	–	–	–	–
2-Ketogluconate K	+	–	+	+
5-Ketogluconate K	–	–	–	–

Table S4. Cellular fatty acid profiles of the strains HO-A22^T, '*E. canadensis*' SHC 2-14, *E. adhaerens* A^T, and *E. morelensis* Lc04^T.

FATTY ACIDS*	HO-A22^T	'*E. canadensis*' SHC 2-14	*E. adhaerens* A^T	*E. morelensis* Lc04^T
$C_{14:0}$ 3-OH	7.5	3.6	–	4.8
$C_{16:0}$ 3-OH	–	–	8.5	–
$C_{16:1}$	4.8	7.2	2.6	8.2
$C_{16:0}$	11.1	10.5	6.9	8.7
$C_{17:1}$	4.6	–	0.3	0.7
$C_{17:0}$	0.4	0.2	–	0.4
$C_{18:0}$ 3-OH	1.1	–	1.9	0.6
$C_{18:1}$	66.8	76.1	75.9	73.7
$C_{18:0}$	3.3	2.4	3.5	2.7
Others	0.4	–	0.4	0.2

*The values are percentages (w/w) of total fatty acids.
